# miR-335-3p attenuates transforming growth factor beta 1-induced fibrosis by suppressing Thrombospondin 1

**DOI:** 10.1371/journal.pone.0311594

**Published:** 2024-10-07

**Authors:** Dong-Hee Han, Min Kyoung Shin, Jung-Suk Sung, Min Kim

**Affiliations:** Department of Life Science, Biomedi Campus, Dongguk University-Seoul, Goyang-si, Gyeonggi-do, Korea; Jordan University of Science and Technology Faculty of Medicine, JORDAN

## Abstract

Pulmonary fibrosis is characterized by excessive extracellular matrix (ECM) accumulation caused by detrimental stimuli. The progressive impairment in lung functions is chronic and highly fatal, presenting itself as a global health challenge. Because of the lack of efficacious treatments, the underlying mechanism should be investigated. The progression of fibrosis involves transforming growth factor-beta 1 (TGF-β1), which accelerates ECM production via epithelial–mesenchymal transition and cell invasion. As microRNAs (miRNAs) serve as regulators of disease development and progression, this study aimed to investigate the interaction of miRNAs and target genes that could contribute to pulmonary fibrosis when exposed to TGF-β1. Differentially expressed mRNA and miRNA were identified in respiratory epithelial cells via transcriptome analysis by using the constructed TGF-β1-induced fibrosis model. Our results revealed a significant increase in the expression of thrombospondin 1 (THBS1), which participates in TGF-β1 activation, where THBS1 was identified as a core gene in protein interactions analyzed through bioinformatics. The expression of miR-335-3p, which targets 3ʹ-UTR of THBS1, substantially decreased upon TGF-β1 treatment. The TGF-β1 downstream signal was suppressed by inhibiting the interaction between TGF-β1 and THBS1, consequently alleviating fibrosis. When the miR-335-3p mimic was transfected in TGF-β1-treated respiratory epithelial cells, THBS1 and fibrosis markers were downregulated, while the introduction of miR-335-3p inhibitor exhibited a reverse phenomenon. Our findings demonstrated that TGF-β1 exposure to respiratory epithelial cells led to a decrease in miR-335-3p expression, resulting in the upregulation of THBS1 and ultimately exacerbating fibrosis. This study provides insights into TGF-β1-induced pulmonary fibrosis, suggesting new therapeutic targets and mechanisms.

## Introduction

Pulmonary fibrosis, which is characterized by the excessive deposition of extracellular matrix (ECM), is a reparative response to tissue damage that results in a chronic decline in organ function [1]. This pathological condition is induced by various factors, such as drug-induced injury, traumatic injury, and infection; it remains a substantial global health challenge [2,3]. Among various organ fibrosis, pulmonary fibrosis is extensively studied because of its life-threatening nature, and patients have an average life expectancy of fewer than 5 years after diagnosis if they are left untreated [4,5]. More than 40,000 new cases of pulmonary fibrosis are diagnosed annually, which is expected to further exacerbate as the elderly population increases worldwide [6]. Additionally, pulmonary fibrosis is associated with other severe diseases, such as emphysema, pulmonary hypertension, and lung cancer, thus prompting comprehensive investigations into its fundamental mechanisms [7–9]. Despite the diverse research efforts, effective therapies for the complete resolution of pulmonary fibrosis are lacking because the exact etiology remains unclear [10–12]. Hence, in-depth research should be performed to identify the fundamental molecular mechanisms that modulate pulmonary fibrosis.

Transforming growth factor-beta 1 (TGF-β1) has raised much attention in fibrosis research because it has been identified as a key factor in the progression of fibrosis [1,13,14]. TGF-β1 is a major regulator of various cellular processes, including cell development, differentiation, recovery, and epithelial–mesenchymal transition (EMT), where it activates intracellular signaling through transforming growth factor β receptor (TβR) and SMAD3 protein [15,16]. Severe tissue stimulation and damage can lead to abnormal expression and increased activation of TGF-β1. In turn, these phenomena induce the differentiation of epithelial cells into mesenchymal cells and fibroblasts via downstream signals; as a result, ECM proteins, such as fibronectin and collagen, excessively accumulate [17,18]. Therefore, determining how TGF-β1 is regulated and activated in fibrosis can contribute to developing treatments for fibrosis.

Thrombospondin 1 (THBS1), one of the evolutionarily conserved secreted proteins, plays various roles, including fibrous tissue connection and vascular regeneration [19,20]. It has several binding sites and can activate various factors, such as fibronectin 1, TGF-β1, and Cluster of Differentiation 47 [21]. It can bind to latent TGF-β1 and convert latent TGF-β1 into active TGF-β1, thereby participating in TGF-β1 signaling activity [22]. Therefore, THBS1 is a potentially important target in TGF-β1-induced fibrosis, and elucidating the THBS1/TGF-β1 regulatory mechanism may contribute to fibrosis treatment [23].

MicroRNAs (miRNAs) are a class of 18–22 nt long RNAs expressed in various species and have a critical regulatory role in a wide range of biological processes [24,25]. By binding to the 3ʹ-untranslated region (UTR) of mRNA, miRNAs can inhibit protein expression in a post-transcriptional manner, causing mRNA degradation or translation suppression [26,27]. Given that miRNAs with protein-regulating functions can act as crucial mediators in disease development, they can be utilized as targets for pulmonary fibrosis [28]. For example, miR-301a targets tuberous sclerosis 1 (TSC1) and induces fibrosis by activating the TSC1/mammalian target of rapamycin (mTOR) signaling [29–32]. Cong-Jie Wang and their colleagues [32] showed that miR-184 can inhibit fibrosis by regulating the TGF-β1-phosphoinositide 3-kinase (PI3K)-protein kinase B (Akt) pathway and targeting mothers against decapentaplegic homolog (SMAD) 2 and Akt.

Previous studies suggested that pulmonary fibrosis may be induced by interactions between TGF-β1 and various factors. Since the dysregulation of gene expression caused by miRNAs can cause the onset of certain diseases, the correlation between miRNAs and specific diseases should be analyzed. Therefore, our study aims to identify the underlying mechanism by which miRNA regulates TGF-β1-induced fibrosis through transcriptome and experimental analysis.

## Material and methods

### Cell culture and treatment

Human lung adenocarcinoma cells (A549) and human bronchial epithelial cells (BEAS-2B) were acquired from the American Type Culture Collection (ATCC, Manassas, VA, USA). The cells were cultured in Roswell Park Memorial Institute Medium (RPMI 1640, Welgene, Daegu, Republic of Korea) supplemented with 10% fetal bovine serum (FBS, Gibco, Grand Island, NY, USA), 100 U/mL penicillin and streptomycin (Welgene, Daegu, Republic of Korea), and 1 mM sodium pyruvate (Welgene, Daegu, Republic of Korea) in a humidified incubator with 5% CO_2_ at 37°C. The cells were initially cultured in 150 mm^2^ dishes; upon reaching 80% confluency, they were seeded into 6-well plates at a density of 20 × 10^4^ cells per well for experimentation. TGF-β1 was purchased from Sigma-Aldrich and stored at -80°C for further use. Various TGF-β1 concentrations (0.1–10 ng/mL) were administered for 2 days after the cells stabilized for 24 h to construct the fibrosis model. For analysis, miRNA mimics and inhibitors were transfected into TGF-β1-treated and untreated groups for 48 h. A THBS1 inhibitor LSKL (MedChemExpress USA, Middlesex, NJ, USA) was simultaneously added at 20 μM in the TGF-β1-treated and untreated groups and cultured for 48 h.

### Cell viability assay

The cell survival rate was determined to assess the toxic effect of TGF-β1 on cells. A549 and BEAS-2B cells were seeded into a 96-well plate at a density of 1 × 10^4^ cells/well. After 48 h of treatment with TGF-β1, the culture medium was aspirated, and the cells were incubated with RPMI 1640 medium containing Quanti-Max WST-8 Cell Viability Assay Solution (WST-8 Solution, Biomax, Seoul, Republic of Korea) at 37°C. After 30 min of incubation, absorbance was measured at 450 nm by using a Sunrise™ Absorbance microplate reader (TECAN, Männedorf, Switzerland).

### RNA isolation

Before the experiment, cells were treated with TGF-β1 for 48 h. Afterward, the cells intended for use were washed twice with phosphate-buffered saline (PBS). Total RNA was then extracted using TRIzol reagent (Invitrogen, Waltham, MA, USA) in accordance with the manufacturer’s instructions. The isolated RNA was dissolved in RNase-free water, and its quality was assessed through agarose gel electrophoresis at 50 V for 30 min. Subsequently, the concentration and purity of the total RNA were determined using a Nanodrop-2000 spectrophotometer (Thermo Fisher Scientific, Waltham, MA, USA). The samples with absorbance readings of 1.8 or higher at 260 and 280 nm were considered to have suitable concentrations for use in the experiment.

### Quantitative real-time polymerase chain reaction (qRT-PCR)

qRT-PCR was conducted and analyzed to validate the fibrosis model. Initially, 2000 ng of isolated RNA was reverse-transcribed into cDNA by using M-MLV reverse transcriptase (ELPIS-BIOTECH, Daejeon, Republic of Korea). For qRT-PCR, filtered deionized water, SYBR Green PCR master mix (KAPA Biosystems, Wilmington, MA, USA), and a real-time PCR detection system (BIO-RAD, Hercules, CA, USA) were utilized. The following reaction conditions were applied: an initial denaturation step at 95°C for 5 min, followed by denaturation at 95°C for 10 s, annealing at 60°C for 30 s, and extension at 72°C for 30 s. This cycle was repeated 50 times to ensure adequate amplification. GAPDH was used as a reference gene for relative comparison, and the expression of other genes was normalized to GAPDH levels. The following primers were used in this study: GAPDH (Forward: TATGACAACAGCCTCAAGAT/ Reverse: GAGTCCTTCCACGATACC), N-cadherin (Forward: CCTCCAGAGTTTACTGCCATGAC/ Reverse: GTAGGATCTCCGCCACTGATTC), Fibronectin (Forward: ACAACACCGAGGTGACTGAGAC/ Reverse: GGACACAACGATGCTTCCTGAG), Col1a1 (Forward: ACTACCTCGTTCTTGTCTT/ Reverse: CCCACCCATCACATAGAT).

### Western blotting

Protein expression levels in cells were evaluated using western blot analysis. The cells were lysed with RIPA buffer (Bio Solution, Seoul, Republic of Korea) supplemented with protease inhibitor cocktail and phosphatase inhibitor cocktails 2/3 (Sigma-Aldrich, St. Louis, MO, USA). After cell lysis, total protein was isolated through centrifugation at 25,000 × *g* for at 4°C 15 min. Subsequently, protein concentration was determined using the BCA protein assay kit (Thermo Fisher, Waltham, MA, USA) in accordance with the manufacturer’s protocol. Afterward, 21 μg of protein per sample were separated through 10% sodium dodecyl sulfate-polyacrylamide gel electrophoresis (SDS-PAGE), and the separated proteins were transferred onto polyvinylidene difluoride membranes (Millipore, Burlington, MA, USA). The membrane was blocked with 5% skim milk at room temperature for 50 min to minimize non-specific binding. The membranes were then incubated with primary antibodies diluted in a 2.5% bovine serum albumin (BSA) solution containing sodium azide (Sigma-Aldrich Chemical, St. Louis, MO, USA) at 4°C overnight. Secondary antibodies were diluted in 1% skim milk and incubated at room temperature for 45 min. The target protein bands were visualized using ECL Plus Western blotting detection reagent (Amersham Bioscience, Buckinghamshire, UK), and images were captured using the Bio-Rad ChemiDoc system. β-actin was used as a loading control for all target proteins; the degree of protein activation was calculated by comparing the phosphorylated proteins with their total forms. The following antibodies were used in this study: p-SMAD3 (sc-517575), SMAD3 (sc-101154), vimentin (sc-6260), β-actin (sc-47778), m-IgG_κ_ BP-HRP (sc-516102), m-IgG_1_ BP-HRP (sc-525408), and m-IgG_Fc_ BP-HRP (sc-5254090), anti-fibronectin antibody (MA5-11981) and THBS1 (MA5-13398), antibodies targeting α-SMA (19245s), anti-rabbit IgG, and HRP-linked antibody (7074s), anti-collagen1 antibody (Ab34710) and goat anti-rabbit IgG Alexa 488 (ab181448), and anti-N-cadherin antibody (66219–1).

### 3ʹ-mRNA quantification sequencing (3ʹ-mRNA Quan-Seq)

3ʹ-mRNA quantification sequencing was conducted by E-Biogen Inc. (Seoul, Republic of Korea). Before the experiment, cells were treated with TGF-β1 for 48 h. Total RNA was isolated, and libraries were prepared using the QuantSeq 3ʹ-mRNA-Seq Library Prep Kit FWD for Illumina (Lexogen, Vienna, Austria) in accordance with the manufacturer’s instructions. Separate mRNA and miRNA libraries were constructed for A549 and BEAS-2B samples. The resulting libraries were analyzed, quantified, and visualized through Excel-based differentially expressed gene analysis (ExDEGA, E-Biogen, Inc., Seoul, Republic of Korea).

### Bioinformatics studies on the transcriptome data

Bioinformatics studies on the transcriptome data were conducted in accordance with the established protocols from previous studies [33]. Differentially expressed genes (DEGs) were identified using the criteria of fold change ≥ 2 and normalized log_2_ data ≥ 7. Subsequently, gene ontology (GO) analysis was performed on genes showing consistent changes in the expression in both cell lines. Gene functional classification was achieved using DAVID (https://david.ncifcrf.gov/, accessed on August 9, 2023). Hierarchical clustering was applied to validate the expression similarity among genes within the top 10 functional groups in GO analysis. Protein–protein interactions were assessed using STRING database version 11.5 (http://string-db.org/, accessed on September 7, 2023), and node rankings within the network were confirmed using the Cytohubba application. Metrics such as Maximal Clique Centrality (MCC), Maximum Neighborhood Component (MNC), and DEGREE were utilized to prioritize interactions and select key factors. For miRNA analysis, miRNAs with significantly altered expression in both cell lines were identified. Subsequently, miRNAs targeting the selected mRNAs were predicted using the MicroRNA Target Prediction Database (https://mirdb.org/, accessed on October 2). Hierarchical clustering was then used to visualize the similarity of miRNA expression. The binding sites between miRNAs and target mRNAs were confirmed using TargetScanHuman version 7.1 (https://www.targetscan.org/vert_71/, accessed October 7).

### miRNA transfection

A549 and BEAS-2B cells were seeded into 6-well plates at a density of 20 × 10^4^ cells/well by using RPMI 1640 medium supplemented with 10% FBS but without antibiotics. Simultaneously, the mimics and inhibitors of miR-335-3p were transfected into the cells by using Lipofectamine 3000 (Invitrogen, Waltham, MA, USA) in accordance with the manufacturer’s instructions. Briefly, they were transfected by mixing the culture medium with Lipofectamine 3000 and miRNA at a final concentration of 25 nM. The transfection mixture and TGF-β1 were then added to the cells and incubated for 48 h before they were used for analysis. The information on the utilized miRNAs is presented in Table 1.

**Table 1:** Information on miR-335-3p mimic and inhibitor.

miRNA	Sequence	Cat. No
**miR-335-3p mimic**	**UUUUUCAUUAUUGCUCCUGACC**	**MCH01884**
**miR-335-3p inhibitor**	**GGUCAGGAGCAAUAAUGAAA**	**HY-RI00694**

### Immunocytochemistry (ICC) staining

For this experiment, a 22 mm × 22 mm cover slide was placed in each well of a 6-well plate and coated with poly-L-lysine before the cells were seeded. A549 and BEAS-2B cells were transfected with miRNA and treated with TGF-β1 in the 6-well plate and then cultured for 48 h. After incubation, the cells were washed with PBS and fixed with 4% formaldehyde for 10 min. They were permeabilized by treating with 0.25% TritonX-100 for 5 min and blocked with 1% bovine serum albumin. Subsequently, the target factors were sequentially stained using primary and secondary antibodies, and the nucleus was stained with DAPI. Fluorescence images were captured using a confocal microscope (Carl Zeiss, Oberkochen, Germany) and quantified using Image J software. The protein expression level was quantified through the ratio of the fluorescence intensity of intracellular factors to the fluorescence intensity of the nucleus.

### Statistical analysis

The experiment was conducted in triplicate. Data were statistically analyzed and presented as mean ± standard error of the mean (SEM). The significance of the results was evaluated using one-way ANOVA followed by Tukey’s post-hoc analysis. Graphs and statistical analyses were generated using GraphPad Prism 5.0 (GraphPad Software Inc., San Diego, CA, USA). Data with p < 0.05 were considered statistically significant.

## Results

### Validation of EMT and ECM accumulation in TGF-β1-induced pulmonary fibrosis model

**Fig 1:**
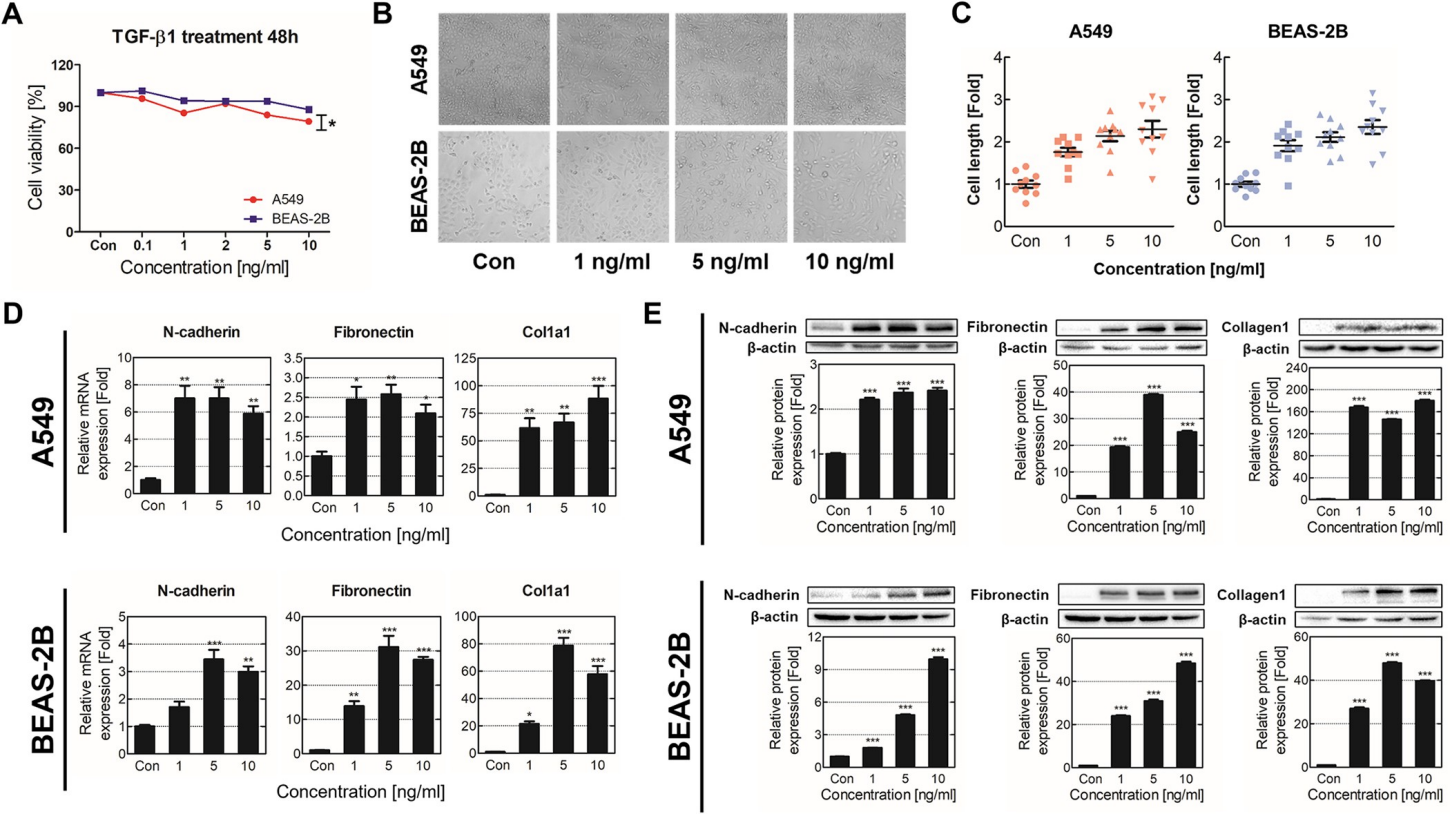
TGF-β1-induced fibrosis is validated in respiratory epithelial cells. **(A)** Cell viability of A549 and BEAS-2B cells was assessed following treatment with various concentrations of TGF-β1 for 48 h. **(B, C)** Morphological changes, including shape and length, were observed in respiratory epithelial cells after 48 h of treatment with TGF-β1. **(D, E)** The mRNA and protein expression levels of N-cadherin, collagen1, and fibronectin were evaluated in A549 and BEAS-2B cells after exposure to TGF-β1 for 48 h. The control group is the TGF-β1 untreated group, and all experiments were performed at least three. *p < 0.05, **p < 0.01, and ***p < 0.001 indicate significance compared with the control group; Con: Control group.

To establish a pulmonary fibrosis model, we first determined the TGF-β1 concentration for treating the respiratory epithelial cell lines A549 and BEAS-2B by evaluating cell viability. After exposure to TGF-β1 concentrations ranging from 0.1 ng/mL to 10 ng/mL for 48 h, cell viability was assessed using WST-8 assay. No significant changes in cell viability were observed within the range of 0.1–5 ng/mL treatment concentrations, while cell viability significantly decreased in A549 cells when treated with 10 ng/mL TGF-β1 (Fig 1A). Cell morphology was examined to confirm the induction of fibrosis upon TGF-β1 treatment (Fig 1B). When exposed to TGF-β1, the cells had an elongated shape, and their length increased in a dose-dependent manner (Fig 1C). Subsequently, the expression of representative fibrosis marker genes was evaluated. The expression of the EMT marker N-cadherin increased in the A549 and BEAS-2B cell lines; likewise, the mRNA and protein expression levels of the ECM proteins fibronectin and collagen1 increased (Fig 1D and 1E). These results indicated that TGF-β1 treatment induced fibrosis in both cell lines. As 10 ng/mL TGF-β1 induced cell toxicity, 5 ng/mL TGF-β1, which caused significant changes in fibrotic characteristics, was used in further analysis.

### Identification of core genes associated with pulmonary fibrosis through transcriptome analysis

**Fig 2:**
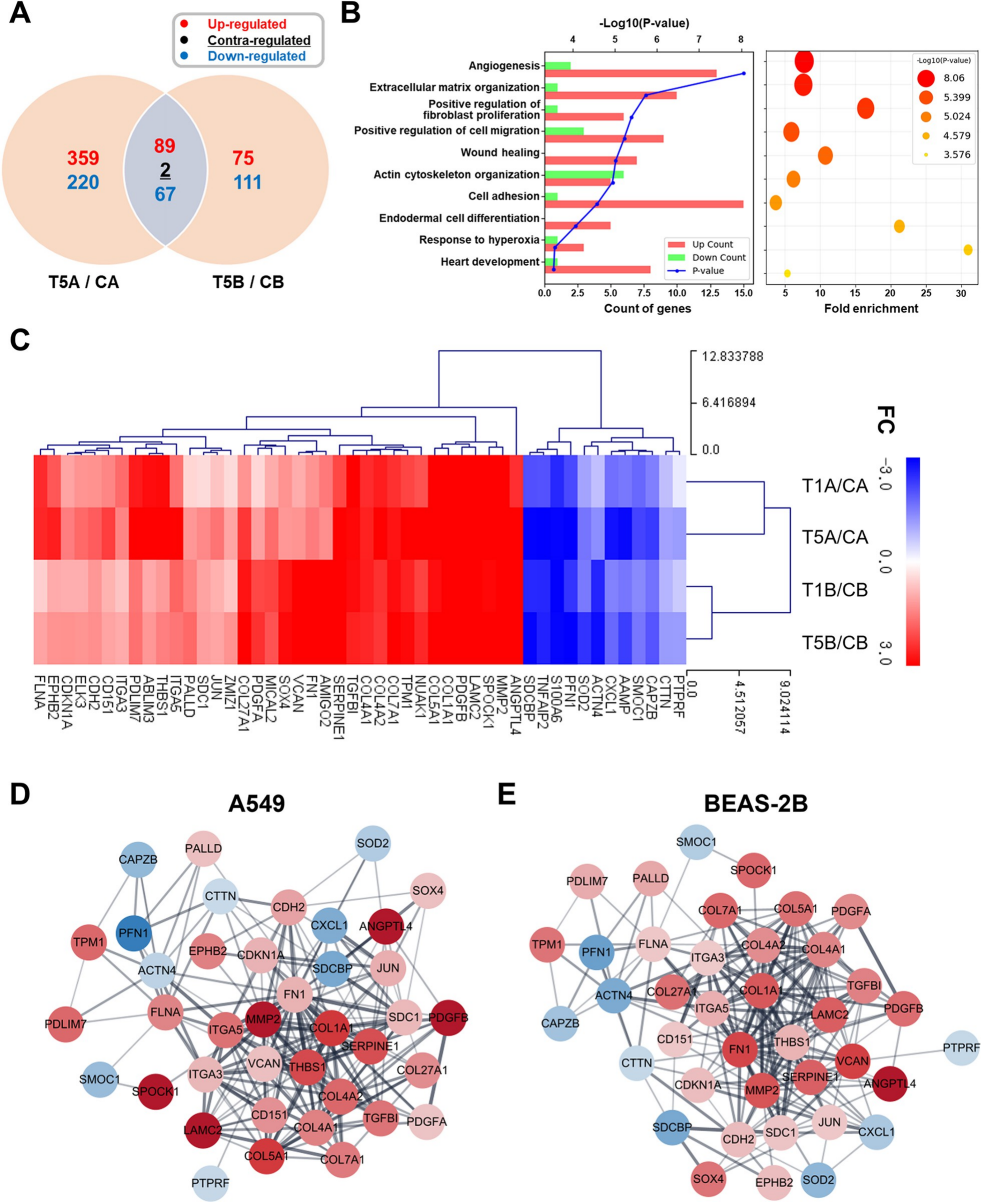
Key biological processes and genes are identified in TGF-β1-induced fibrosis via transcriptome analysis. After treatment with 1 and 5 ng/mL TGF-β1 for 48 h, mRNA transcriptome analysis was performed. **(A)** Differentially expressed genes (DEGs; ≥2 folds, ≥7 normalized data value) significantly altered by TGF-β1 treatment were depicted by a Venn diagram. **(B)** Gene ontology analysis was conducted on 156 DEGs with consistent expressional changes in both cell types, resulting in 48 DEGs associated with the top 10 biological processes. **(C)** Hierarchical clustering was performed to illustrate the expression patterns of the 48 chosen DEGs. **(D, E)** Protein–protein interaction was analyzed on the 48 selected DEGs; red and blue represented the increased and decreased expression levels, respectively, in response to TGF-β1 treatment. CA: Control group of A549; CB: Control group of BEAS-2B; T1A: 1 ng/mL TGF-β1-treated group of A549; T1B: 1 ng/mL TGF-β1-treated group of BEAS-2B; T5A: 5 ng/mL TGF-β1-treated group of A549; T5B: 5 ng/mL TGF-β1-treated group of BEAS-2B; FC: Fold change.

To investigate how TGF-β1 regulates the expression of genes related to pulmonary fibrosis, we performed bioinformatics studies on the transcriptome data after the treatment with TGF-β1 for 48 h. We analyzed and compared the changes in gene expression between the control group and TGF-β1-treated groups. Specifically, genes with a fold change (FC) of ≥2 and a normalized data (ND) value of ≥7 were selected as differentially expressed genes (DEGs). The TGF-β1 treatment affected the expression of 737 and 344 genes in A549 and BEAS-2B cells, respectively. Among them, 158 genes displayed a significant differential expression in both cell lines (Fig 2A). We analyzed gene ontology (GO) and gene fold enrichment of 156 concertedly upregulated or downregulated genes (Fig 2B). Our analysis revealed that DEGs were associated with biological processes (BPs), including angiogenesis, ECM organization, fibroblast proliferation, cell migration, and wound healing. Subsequently, we performed hierarchical clustering and protein–protein interaction (PPI) analyses on 48 genes belonging to the top 10 BPs identified by GO analysis (Fig 2C–2E). We observed that the expression of the genes associated with TGF-β1-induced fibrosis increased, which was indicated by their expressional correlations and interactions. To prioritize the genes within their interactions, we applied three protein network analysis algorithms: Maximum Neighborhood Component (MNC), Density of Maximum Neighborhood Component (DMNC), and DEGREE (S1 Table and S1 Fig). Consistently, we identified eight genes belonging to the top 10 core genes across the three analyses (Table 2). These results indicated that ECM-related genes and THBS1 are concurrently upregulated during fibrosis. Therefore, THBS1 likely plays a specific role in fibrosis progression and development.

**Table 2:** Selection of key genes based on protein–protein interaction analysis.

Gene name	String ID	Ranking method	Fold change (5 μM)		Gene description
			A549	BEAS-2B	
FN1	9606.ENSP00000346839	MCC, MNC, Degree	2.930	15.846	Fibronectin 1 (2477 aa)
THBS1	9606.ENSP00000260356	MCC, MNC, Degree	14.675	2.844	Thrombospondin 1 (1170 aa)
COL1A1	9606.ENSP00000225964	MCC, MNC, Degree	19.434	11.671	Collagen alpha-1(I) chain (1464 aa)
MMP2	9606.ENSP00000219070	MCC, MNC, Degree	49.949	12.659	72 kDa type IV collagenase (660 aa)
COL4A1	9606.ENSP00000364979	MCC, MNC, Degree	6.199	5.902	Collagen alpha-1(IV) chain (1669 aa)
COL4A2	9606.ENSP00000353654	MCC, MNC, Degree	9.213	5.167	Collagen alpha-2(IV) chain (1712 aa)
ITGA5	9606.ENSP00000360882	MCC, MNC, Degree	7.884	3.043	Integrin alpha-5 heavy chain (1049 aa)
COL5A1	9606.ENSP00000293379	MCC, MNC, Degree	18.961	8.079	Collagen alpha-1(V) chain (1838 aa)

### Selection of the miRNA regulating the core gene THBS1 in TGF-β1-induced pulmonary fibrosis

**Fig 3:**
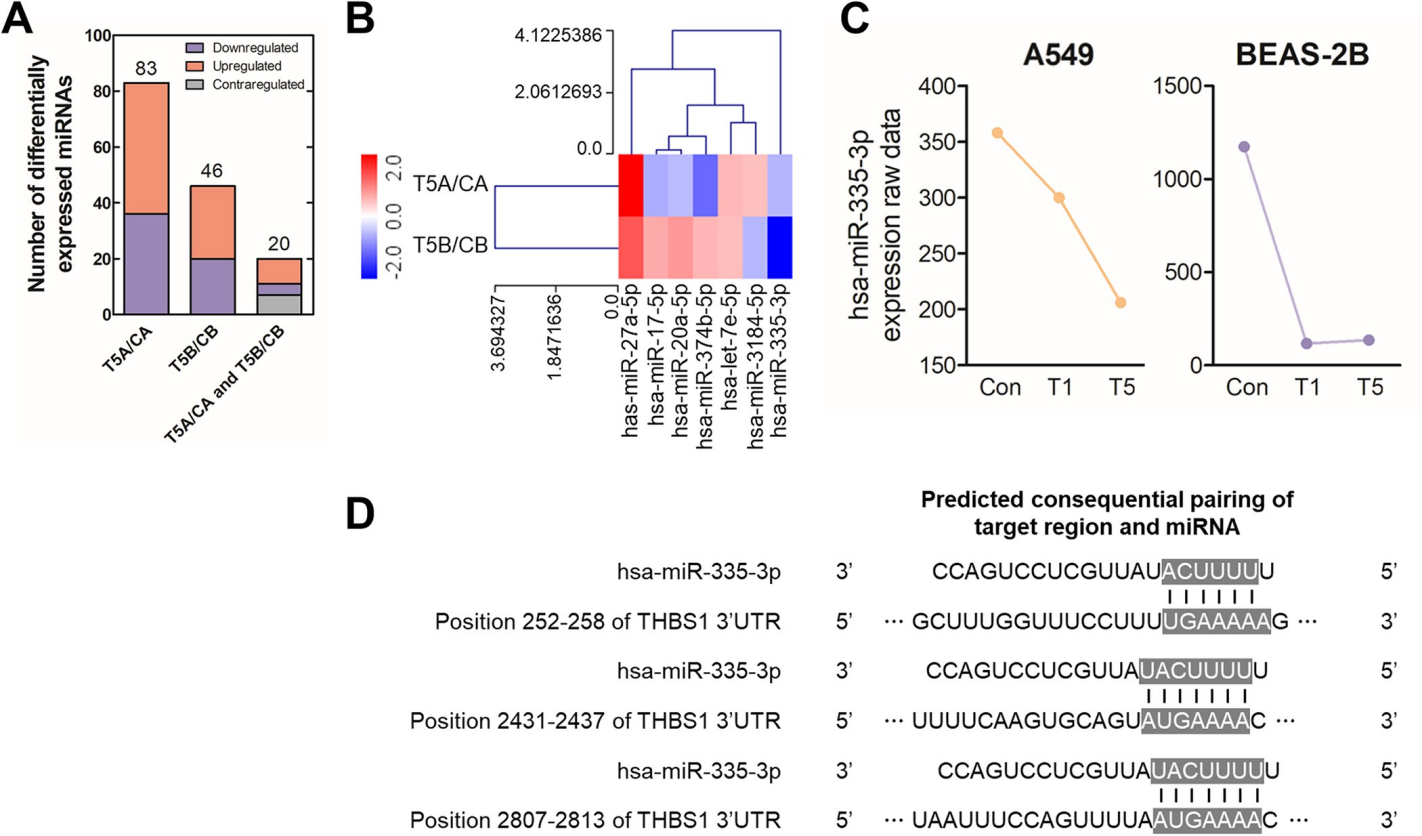
miR-335-3p targeting THBS1 is downregulated in the fibrosis model. Transcriptome analysis on miRNA was conducted after treatment with 1 and 5 ng/mL TGF-β1 for 48 h. **(A)** miRNAs with significantly changed expression following TGF-β1 treatment were identified for each cell type. **(B)** Hierarchical clustering was performed on miRNAs targeting the core genes selected from those showing significant expression changes. **(C)** The expression of miR-335-3p decreased after TGF-β1 treatment in both cell lines. **(D)** The binding site of miR-335-3p is depicted on the 3ʹ-untranslated region of THBS1. Con: Control group (TGF-β1-untreated group); T1: 1 ng/mL TGF-β1-treated group; T5: 5 ng/mL TGF-β1-treated group.

We analyzed the differentially expressed miRNAs to identify those that could regulate the expression of the identified core genes. After TGF-β1 treatment, the expression of 83 and 46 miRNAs significantly changed (FC ≥ 1.4 and ND value ≥ 8) in A549 and BEAS-2B cells, respectively (Fig 3A). From the 20 miRNAs showing expressional changes in both cell lines, we further selected 7 miRNAs that could target eight core genes; among them, miR-335-3p was downregulated in both cell lines (Fig 3B). Notably, the miR-335-3p expression decreased by more than 40% when respiratory epithelial cells were treated with TGF-β1 (Fig 3C). On the basis of the miRDB and TargetScanHuman database, we investigated whether miR-335-3p could target the eight previously selected core genes. We found that three binding sites in the 3ʹ-UTR of THBS1 could be the target of miR-335-3p (Fig 3D). This finding suggested the potential role of miR-335-3p in regulating the biological function of THBS1 in influencing fibrosis.

### Inhibition of THBS1 downregulates TGF-β1 activation and attenuates fibrosis

**Fig 4:**
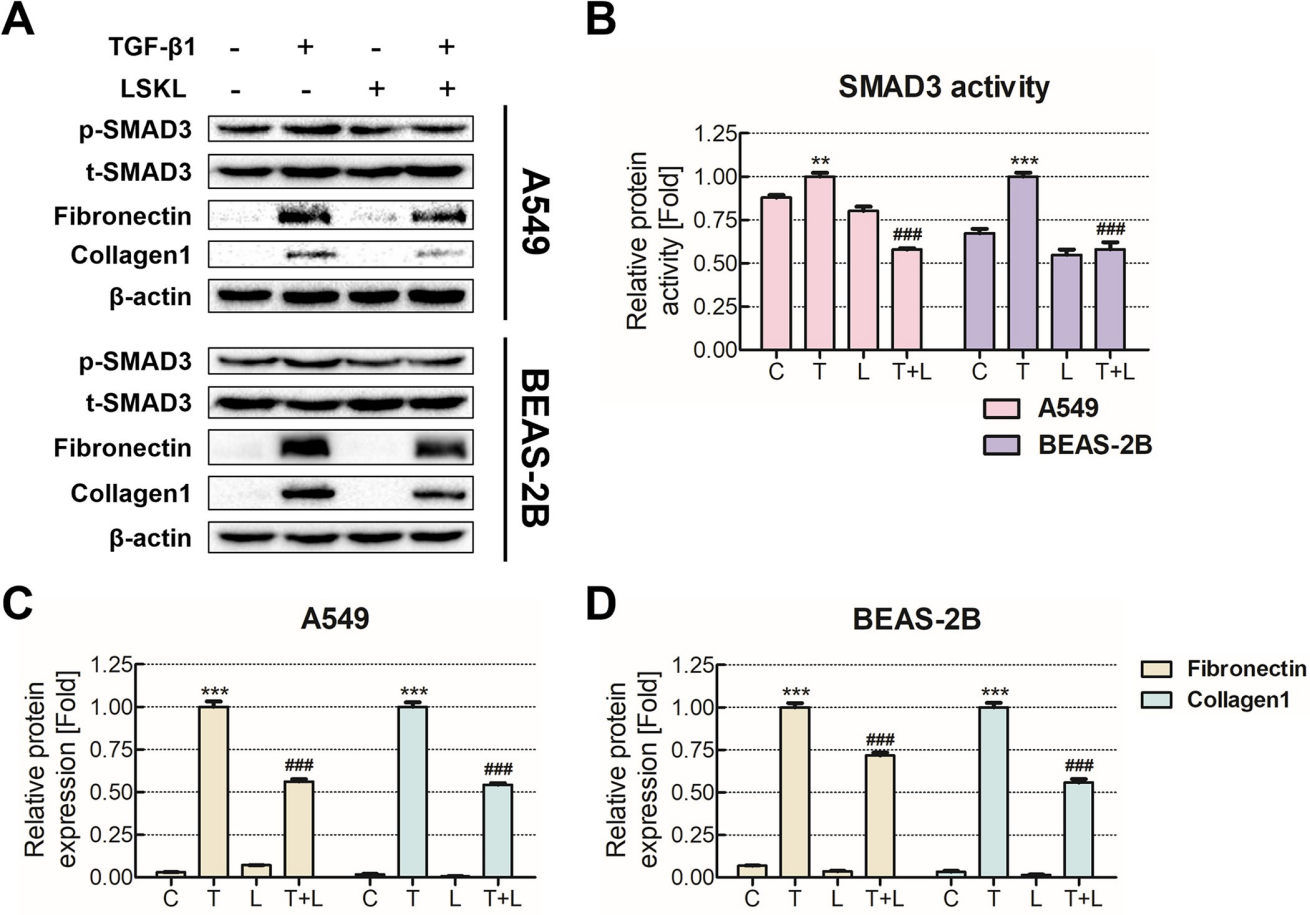
THBS1 inhibition suppresses TGF-β1 activity and downstream signaling. A549 and BEAS-2B cells were treated with 5 ng/mL TGF-β1 and/or 20 μM LSKL for 48 h. **(A)** The expression of proteins associated with TGF-β1 downstream signaling was assessed by western blotting. **(B)** SMAD3 activation in response to TGF-β1 treatment was determined by the ratio of phosphorylated SMAD3 to total SMAD3 expression. **(C, D)** The protein expression of fibrosis-related genes in A549 and BEAS-2B cells was presented using bar graphs. Each experiment was conducted in triplicate. * *p* < 0.05; ** *p* < 0.01; *** *p* < 0.001 compared with the control group.; C: Control group; T: TGF-β1 treated group; L: LSKL-treated group; T+L: TGF-β1- and LSKL-treated group.

To assess the potential role of THBS1 in regulating TGF-β1 signaling and inducing fibrosis, we treated the cells with LSKL, an inhibitor of THBS1 that acts as a TGF-β1 antagonist (Fig 4A). TGF-β1 treatment induced the phosphorylation of SMAD3, a key mediator of TGF-β1 signaling. After the cells were co-treated with TGF-β1 and LSKL, the activation of SMAD3 was attenuated in both cell lines (Fig 4B). Subsequently, we examined the protein expression of fibronectin and collagen1, which are well-known fibrosis markers (Fig 4C and 4D). These marker proteins were significantly reduced in both cell lines when LSKL was co-administered with TGF-β1 compared with TGF-β1 treatment alone. These findings suggested that the modulation of TGF-β1 signaling by THBS1 may influence fibrosis initiation and progression.

### Regulation of fibrosis through the THBS1/miR-335-3p axis

**Fig 5:**
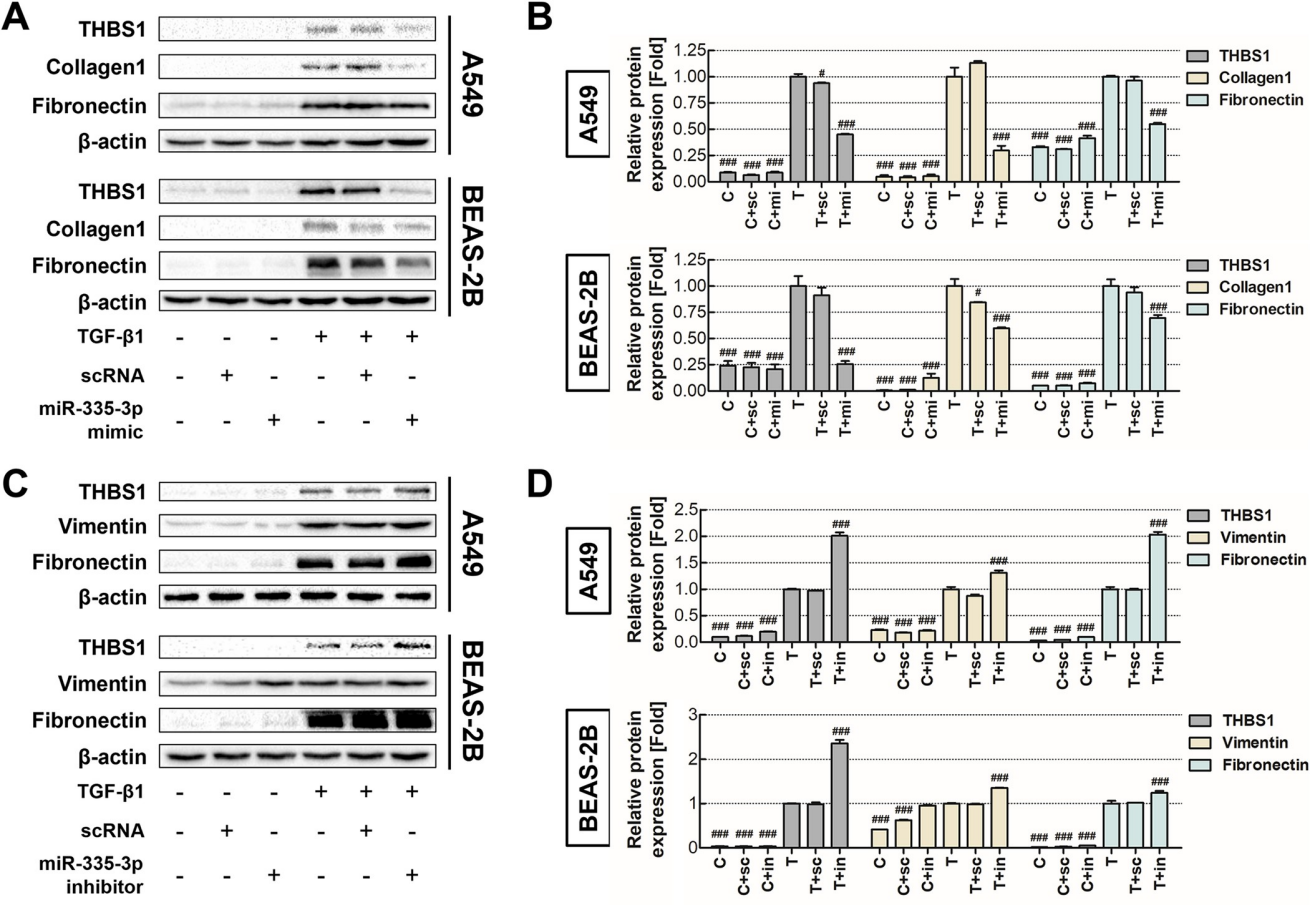
miR-335-3p downregulates THBS1 expression to inhibit fibrosis. The expression of miR-335-3p was altered in A549 and BEAS-2B cell lines to observe the regulatory effect of miR-335-3p on THBS1. **(A, B)** THBS1 expression and fibrosis markers were suppressed following transfection with miR-335-3p mimic. **(C, D)** The upregulation of TBHS1 and fibrotic marker expression were observed after the introduction of miR-335-3p inhibitor. Each experiment was conducted in triplicate. # *p* < 0.05; ## *p* < 0.01; ### *p* < 0.001 compared with the TGF-β1-only-treated group. C: Control group; T: 5 ng/mL TGF-β1-treated group; sc: scrambled RNA-treated group; mi:miR-335-3p mimic-treated group; in: mi:miR-335-3p inhibitor-treated group.

THBS1 can regulate fibrosis by interacting with TGF-β1 and modulating downstream signaling. Thus, we investigated whether miRNAs could affect the onset of fibrosis by modulating THBS1 expression under TGF-β1 induction. To validate the interaction between the THBS1 gene and the selected miR-335-3p, we transfected the cells with miR-335-3p mimics and inhibitors in both cell lines and assessed the expression of THBS1 and fibrosis markers (Fig 5). After the miR-335-3p mimics were introduced, the THBS1 expression significantly decreased by more than 50% in both cell lines compared with that in the TGF-β1-single-treated group; furthermore, the expression of fibrosis markers significantly decreased (Fig 5B). Subsequently, the THBS1 expression increased by approximately twofold after the treatment with miR-335-3p inhibitors; the expression of fibrosis markers also significantly increased (Fig 5D). Consistent with the previous results, our findings showed that miR-335-3p mimics inhibited alpha-smooth muscle actin, an intracellular fibrosis marker (S2 Fig). These results suggested that miR-335-3p could regulate the THBS1 expression and thus participated in TGF-β1-induced pulmonary fibrosis.

## Discussion

Global aging and environmental pollution, such as fine dust, cause chronic damage to the lungs, consequently increasing the incidence of pulmonary fibrosis [6]. Despite the increasing incidence and severity of pulmonary fibrosis, the exact molecular mechanisms underlying pulmonary fibrosis have not been fully explored [34]. Therefore, further research should be performed to completely understand pulmonary fibrosis.

Exposure to detrimental stimuli and subsequent biological responses increase the TGF-β1 expression and secretion in the respiratory system [35,36]. TGF-β1 is involved in the development of fibrosis by promoting myofibroblast transformation and EMT through various signaling pathways, including SMAD3, PI3K, and extracellular signal-regulated kinases [37–39]. Although several therapeutic agents targeting these downstream pathways have been developed, no definitive cure for pulmonary fibrosis is available [40]. Studies on TGF-β1-mediated molecular mechanisms could offer potential insights into treatments for pulmonary fibrosis. In our study, we established a fibrosis model with A549 and BEAS-2B respiratory epithelial cell lines by treating them with TGF-β1. As shown in Fig 1, we assessed the toxicity of TGF-β1 on respiratory cells and observed the morphological changes in the cells after they were treated with 1–10 ng/mL TGF-β1. The evaluation of key fibrotic factors showed that the expression was significantly changed at 5 and 10 ng/mL, indicating that fibrosis was induced in respiratory cells (Fig 1D and 1E). Combining the data on cellular toxicity, morphological changes, and molecular markers, we determined that the optimal TGF-β1 concentration that could induce fibrosis in pulmonary epithelial cells was 5 ng/mL.

On the basis of the constructed fibrosis model, we performed mRNA transcriptome analysis to identify the key factors and molecular pathways involved in TGF-β1-induced fibrosis. The treatment with 5 ng/mL TGF-β1 significantly altered the expression of numerous genes in A549 and BEAS-2B cells (Fig 2A). GO analysis of 156 DEGs with a common pattern of expressional changes in both cell lines revealed that the genes involved in angiogenesis, ECM organization, fibroblast proliferation, and wound healing were upregulated (Fig 2B). These findings suggested that TGF-β1 treatment could induce multiple biological processes that resulted in fibrosis. To identify the core genes mainly participating in TGF-β1-induced fibrosis, we selected the top 10 GO categories and analyzed the corresponding DEGs. Notably, the THBS1 expression increased in a dose-dependent manner in both cell lines; THBS1 also actively interacted with other DEGs (Fig 2D and 2E). Using three algorithms to assess the identification of functional clusters of protein nodes, the central role of the largest clique in the network, and the number of interacting proteins, we prioritized eight DEGs within the PPI network (Table 2). Among them, THBS1 was shown to rank highly in all algorithms, suggesting that the THBS1 expression was regulated by TGF-β1 exposure and functionally involved in fibrosis induction. THBS1 is closely involved in the activation of TGF-β1 through structural binding, and the increased TGF-β1 activation can affect cell migration, angiogenesis, fibrosis, and other processes [41,42]. However, most studies on THBS1 have focused only on TGF-β1 activation of THBS1, and research on the increase in THBS1 by TGF-β1 and the mechanisms that induce this is still insufficient. Thus, our study aimed to identify the THBS1-related mechanisms affecting TGF-β1-induced pulmonary fibrosis.

miRNAs have been suggested as key regulators in various diseases, including cancer, fibrosis, and immune disorders, which are closely related to pathogenesis and prognosis [43–45]. Accordingly, we postulated that TGF-β1 might induce changes in the mRNA and miRNA expression, regulating the expression of core genes such as THBS1 and ultimately leading to fibrosis. Transcriptome analysis was conducted to evaluate the changes in miRNA expression following TGF-β1 treatment. On the basis of the miRNA expression that significantly changed in both cell lines, we identified seven miRNAs targeting eight core genes from the fibrosis model (Fig 3A and 3B). Specifically, miR-335-3p was drastically downregulated upon TGF-β1 treatment (Fig 3C). Since miRNAs bind to the 3ʹ-UTR of mRNA to suppress its expression, we analyzed the binding sites of miR-335-3p within the 3ʹ-UTR of THBS1 [26,46]. We identified three binding regions of miR-335-3p, suggesting that miR-335-3p could regulate the THBS1 expression (Fig 3D). Thus, we hypothesized that the TGF-β1-induced reduction of miR-335-3p increases the THBS1 protein expression, which in turn enhances the TGF-β1 activation to accelerate fibrosis.

We aimed to assess the functional association between TGF-β1 and THBS1 on lung fibrosis. To investigate this association, we used LSKL, a known TGF-β1 antagonist inhibiting the binding site of THBS1, to determine whether the TGF-β1-induced increase in THBS1 could ultimately influence the fibrotic features of pulmonary epithelial cells. We evaluated the TGF-β1 downstream molecules, such as SMAD3. We observed that SMAD3 activation and fibrosis marker expression significantly decreased (Fig 4). These results implied that the THBS1/TGF-β1 interaction might induce a positive feedback loop, thus enhancing TGF-β1 activation and promoting fibrosis.

To verify the effect of the THBS1/miR-335-3p interaction on fibrosis, we treated the TGF-β1-induced fibrosis model with miR-335-3p mimic and inhibitor. Protein expression analysis showed that the presence or absence of miR-335-3p influenced the THBS1 expression and subsequently altered the expression of fibrosis markers in both cell lines (Figs 5 and S2). These findings suggested that miR-335-3p could downregulate the expression of THBS1, playing a critical role in the development of TGF-β1-induced fibrosis; it might also serve as a potential therapeutic target.

In this study, we aimed to discover the unknown underlying the change in miRNA expression by TGF-β1 and its correlation with the fibrosis phenotype. To investigate this, we constructed a fibrosis model by using TGF-β1 to simulate the development of fibrosis and investigated the changes in the miRNA/mRNA expression associated with TGF-β1-induced fibrosis. Exposure to TGF-β1 led to morphological and molecular changes in A549 and BEAS-2B pulmonary epithelial cell lines, altering the expressional patterns of mRNAs and miRNAs. Specifically, TGF-β1 exposure caused a substantial increase in the THBS1 expression which was caused by a decrease in the miR-335-3p expression; thus, TGF-β1 signaling was activated, and the expression of fibrosis markers was upregulated. Further study should be conducted to elucidate by which molecular pathway the expression of miR-335-3p is regulated in TGF-β1-induced fibrosis. Moreover, investigating whether the interaction between miR-335-3p and THBS1 acts as a core mechanism in fibrosis caused by factors other than TGF-β1 is expected to be valuable in developing treatments for this typically refractory condition. Our research, for the first time, demonstrated that the interaction between miR-335-3p and THBS1 contributed to the development of TGF-β1-induced fibrosis. This study offered a new approach to investigating the pathogenesis of pulmonary fibrosis and described potential therapeutic targets.

## Supporting information

S1 Table Selection of core genes through PPI algorithm. (DOCX)

S2 Table Selection of miRNAs that can target core genes. (DOCX)

S1 Fig Core genes were selected through PPI analysis based on three algorithms. Among 46 DEGs whose expression significantly changed in A549 and BEAS-2B cell lines, the core genes were selected using three algorithms: MCC, MNC, and DEGREE. **(A)** The top 10 genes were prioritized using MCC analysis, which identifies the largest clique within the PPI network and selects the central proteins within the clique. **(B)** The priority of the top 10 genes was evaluated through MNC, which identifies clusters of protein nodes that are more functionally connected to each other and selects the central proteins within the cluster. **(C)** The top 10 genes were prioritized based on DEGREE analysis, which selects core proteins based on the number of interactions proteins have in the network.
(TIF)

S2 Fig miR-335-3p modulates intracellular fibrotic marker expression in respiratory epithelial cells. **(A, B)** Following treatment with TGF-β1 and miR-335-3p mimic transfection in A549 and BEAS-2B cell lines, intracellular expression of the fibrotic marker α-smooth muscle actin (α-SMA) was assessed using fluorescence imaging. Green indicates intracellular expression of α-SMA, whereas blue DAPI staining indicates nuclei. **(C)** The expressional value of intracellular α-SMA was quantified by the ratio of green and blue fluorescence values and was presented as a bar graph. C: Control group; sc: scrambled RNA treated group; mi: miR-335-3p mimic treated group; T: TGF-β1 treated group.
(TIF)

S1 Raw image (PDF)
