# Supplementary material for: miR-335-3p attenuates transforming growth factor beta 1-induced fibrosis by suppressing Thrombospondin 1
Source: PLoS One. 2024 Oct 7;19(10):e0311594. doi: 10.1371/journal.pone.0311594 (PMC11457990; doi:10.1371/journal.pone.0311594)
Supplement: S1 Table — (DOCX) [file pone.0311594.s001.docx]

**S1 Table. Analysis of core genes through three PPI algorithms**

| Top 10 genes ranked  by MCC | | | Top 10 genes ranked  by MNC | | | Top 10 genes ranked  by DEGREE | | |
| --- | --- | --- | --- | --- | --- | --- | --- | --- |
| Gene name | Rank | Score | Gene name | Rank | Score | Gene name | Rank | Score |
| FN1 | 1 | 306102 | FN1 | 1 | 27 | FN1 | 1 | 27 |
| COL1A1 | 2 | 305407 | THBS1 | 2 | 22 | THBS1 | 2 | 22 |
| THBS1 | 3 | 305136 | COL1A1 | 3 | 21 | COL1A1 | 3 | 21 |
| COL4A1 | 4 | 293880 | MMP2 | 3 | 21 | MMP2 | 3 | 21 |
| COL5A1 | 5 | 288120 | COL4A2 | 5 | 17 | COL4A2 | 5 | 17 |
| MMP2 | 6 | 218601 | COL4A1 | 5 | 17 | COL4A1 | 5 | 17 |
| COL4A2 | 7 | 208200 | SERPINE1 | 7 | 16 | SERPINE1 | 7 | 16 |
| ITGA5 | 8 | 171576 | ITGA5 | 7 | 16 | ITGA5 | 7 | 16 |
| TGFBI | 9 | 162000 | COL5A1 | 9 | 15 | COL5A1 | 9 | 15 |
| LAMC2 | 10 | 121032 | CDH2 | 10 | 13 | VCAN | 10 | 13 |
