# Supplementary material for: miR-335-3p attenuates transforming growth factor beta 1-induced fibrosis by suppressing Thrombospondin 1
Source: PLoS One. 2024 Oct 7;19(10):e0311594. doi: 10.1371/journal.pone.0311594 (PMC11457990; doi:10.1371/journal.pone.0311594)
Supplement: S2 Table — (DOCX) [file pone.0311594.s002.docx]

**S2 Table. Selection of miRNAs that can target core genes**

| **miRNA** | **Fold change (5 μM)** | | **Selected targets of miRNA in core genes** |
| --- | --- | --- | --- |
|  | **A549** | **BEAS-2B** |  |
| hsa-let-7e-5p | 1.483 | 1.431 | THBS1, COL1A1, COL4A1, COL4A2 |
| hsa-miR-17-5p | 0.641 | 1.591 | MMP2, COL4A1, COL4A2 |
| hsa-miR-20a-5p | 0.691 | 1.751 | MMP2, COL4A1, COL4A2 |
| hsa-miR-27a-5p | 5.276 | 2.508 | FN1 |
| hsa-miR-374b-5p | 0.444 | 1.500 | COL4A1 |
| hsa-miR-3184-5p | 1.410 | 0.677 | COL1A1, ITGA5 |
| hsa-miR-335-3p | 0.673 | 0.148 | THBS1, COL4A1 |
