# Supplementary figures and images for: miR-335-3p attenuates transforming growth factor beta 1-induced fibrosis by suppressing Thrombospondin 1
Source: PLoS One. 2024 Oct 7;19(10):e0311594. doi: 10.1371/journal.pone.0311594 (PMC11457990; doi:10.1371/journal.pone.0311594)

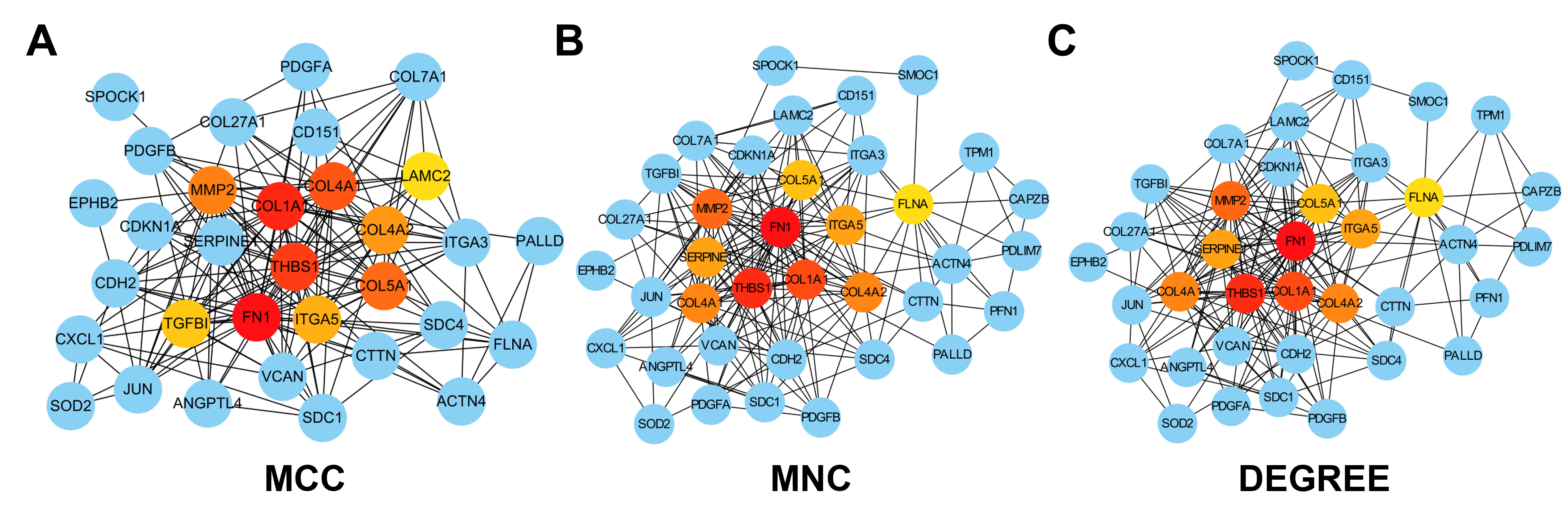

Supplement: S1 Fig — Among 46 DEGs whose expression significantly changed in A549 and BEAS-2B cell lines, the core genes were selected using three algorithms: MCC, MNC, and DEGREE. (A) The top 10 genes were prioritized using MCC analysis, which identifies the largest clique within the PPI network and selects the central proteins within the clique. (B) The priority of the top 10 genes was evaluated through MNC, which identifies clusters of protein nodes that are more functionally connected to each other and selects the central proteins within the cluster. (C) The top 10 genes were prioritized based on DEGREE analysis, which selects core proteins based on the number of interactions proteins have in the network. (TIF) [file pone.0311594.s003.tif]

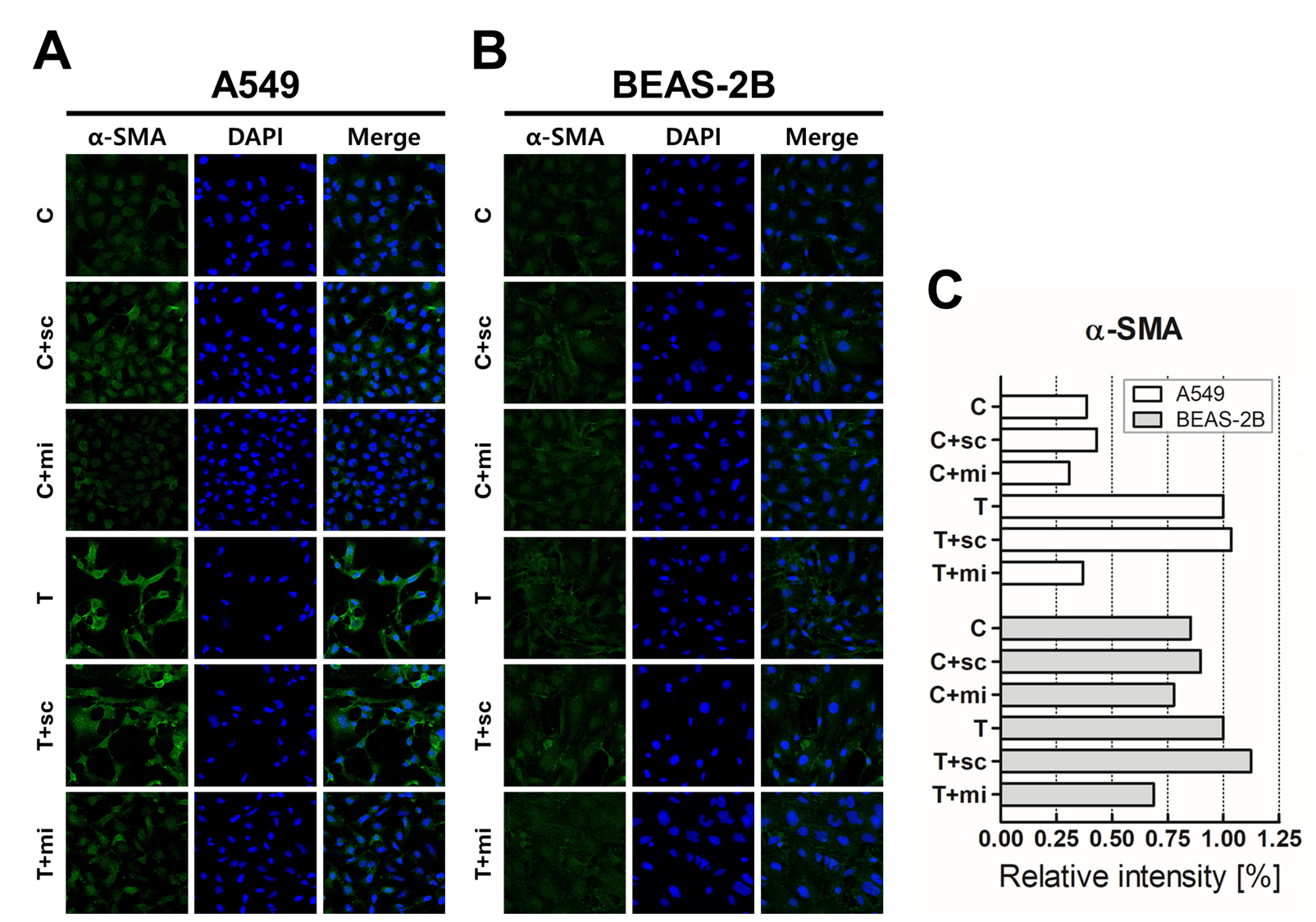

Supplement: S2 Fig — (A, B) Following treatment with TGF-β1 and miR-335-3p mimic transfection in A549 and BEAS-2B cell lines, intracellular expression of the fibrotic marker α-smooth muscle actin (α-SMA) was assessed using fluorescence imaging. Green indicates intracellular expression of α-SMA, whereas blue DAPI staining indicates nuclei. (C) The expressional value of intracellular α-SMA was quantified by the ratio of green and blue fluorescence values and was presented as a bar graph. C: Control group; sc: scrambled RNA treated group; mi: miR-335-3p mimic treated group; T: TGF-β1 treated group. (TIF) [file pone.0311594.s004.tif]
