# Supplementary material for: miR-335-3p attenuates transforming growth factor beta 1-induced fibrosis by suppressing Thrombospondin 1
Source: PLoS One. 2024 Oct 7;19(10):e0311594. doi: 10.1371/journal.pone.0311594 (PMC11457990; doi:10.1371/journal.pone.0311594)
Supplement: S1 Raw image — (PDF) [file pone.0311594.s005.pdf]

miR-335-3p attenuates Transforming growth factor 1-induced fibrosis by suppressing Thrombospondin 1

Western blot raw data

Dong-Hee Han, Min Kyoung Shin, Jung-Suk Sung*, Min Kim*

PM2510

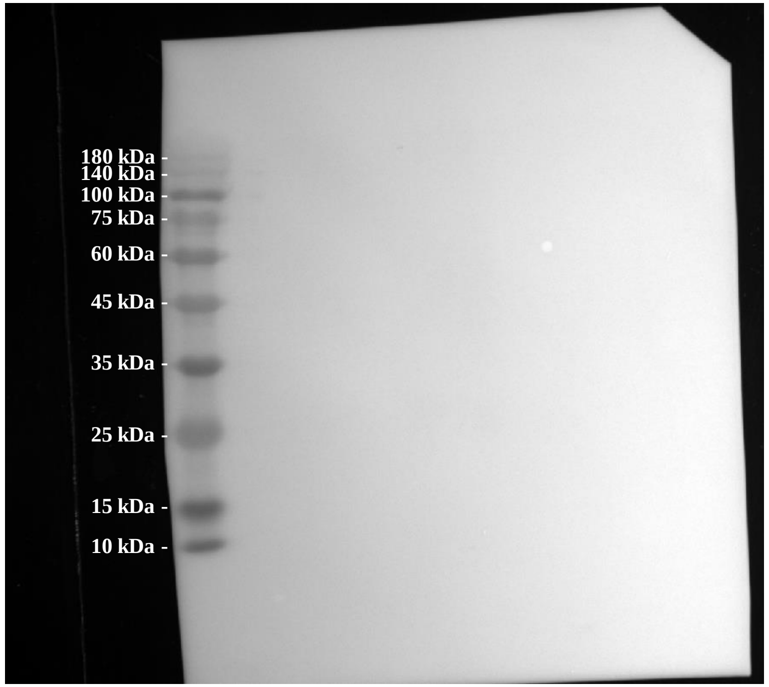

P8500-040

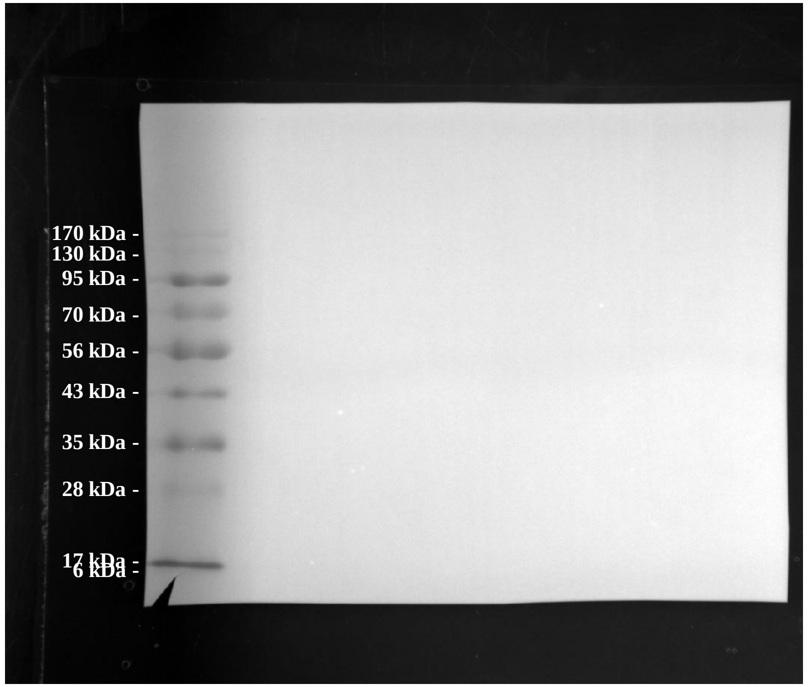

Protein ladder used in this study

Figure 1. raw data

All of our protein quantifications were based on reference genes measured on the same blot.

Figure 1E. A549_N-cadherin (host : Mouse)

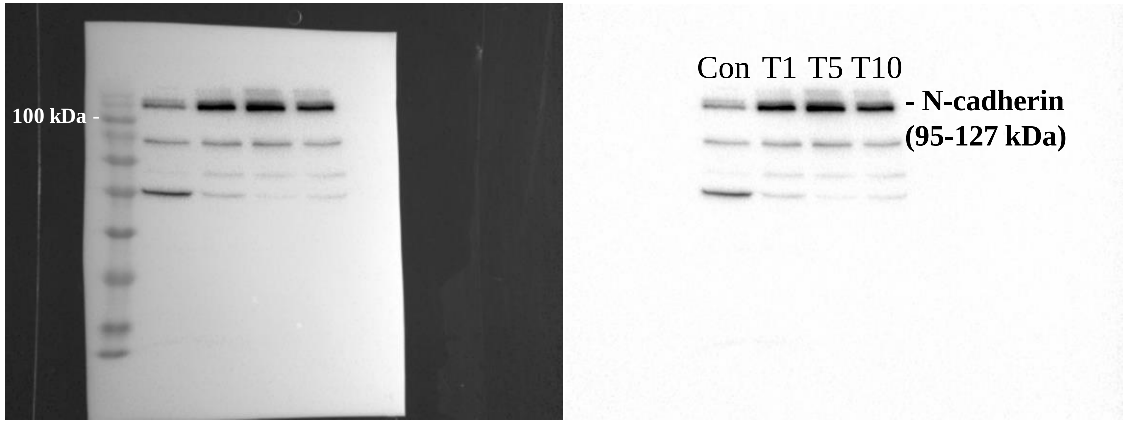

Figure 1E. A549_Fibronectin (host : Mouse)

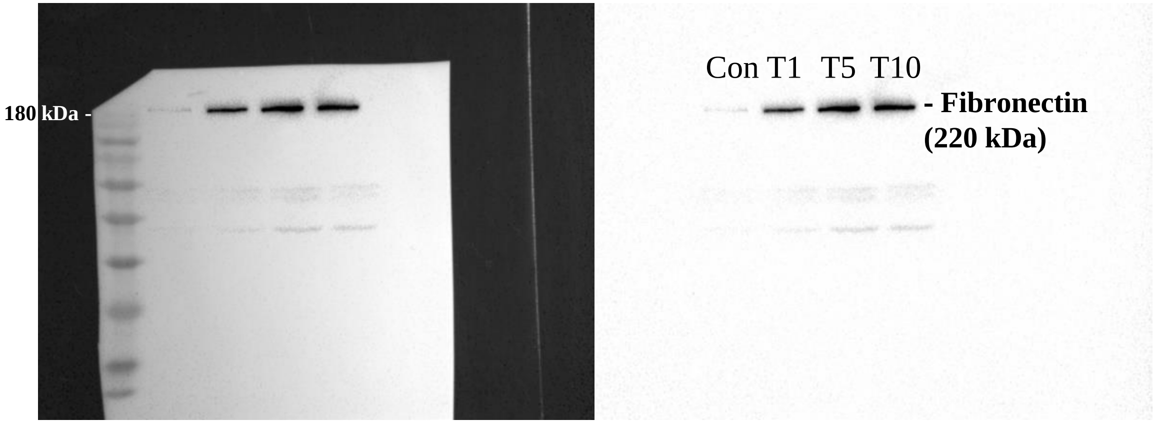

Figure 1E. A549_β-actin (host : Mouse)

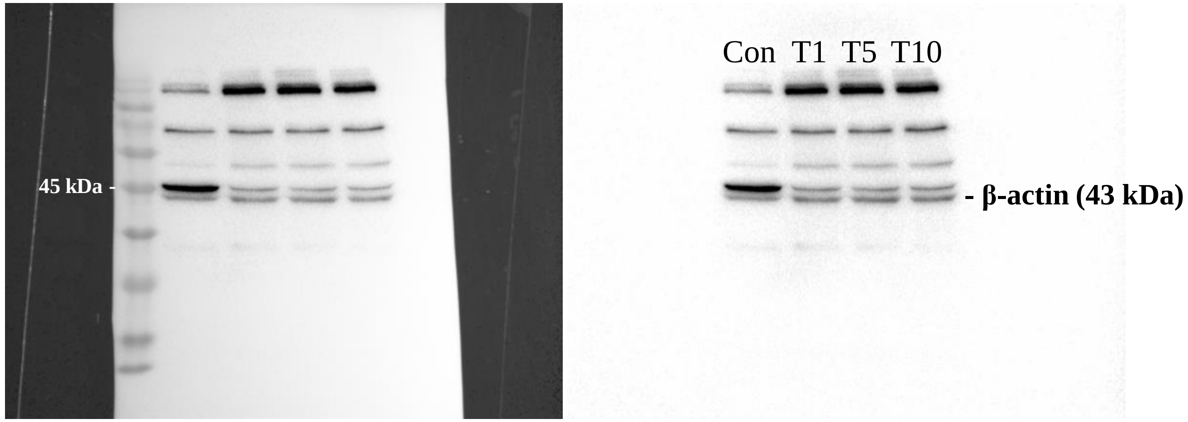

Figure 1E. A549_β-actin (host : Mouse)

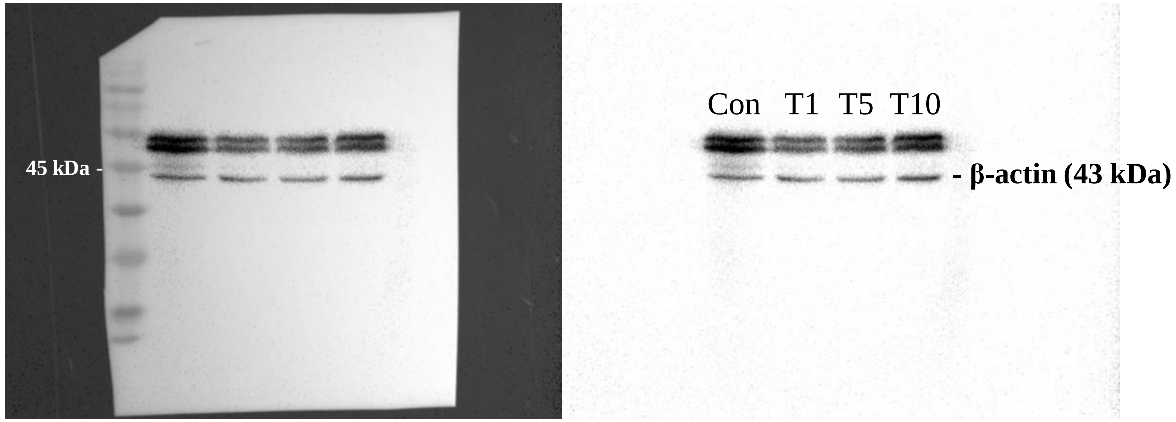

Figure 1. raw data

All of our protein quantifications were based on reference genes measured on the same blot.

Figure 1E. A549_Collagen1 (host : Rabbit)

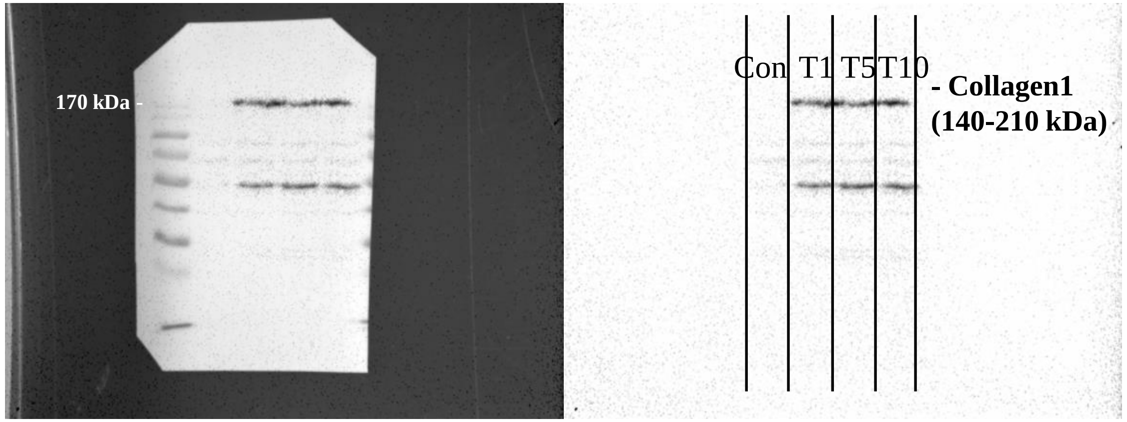

Figure 1E. BEAS-2B_N-cadherin (host : Mouse)

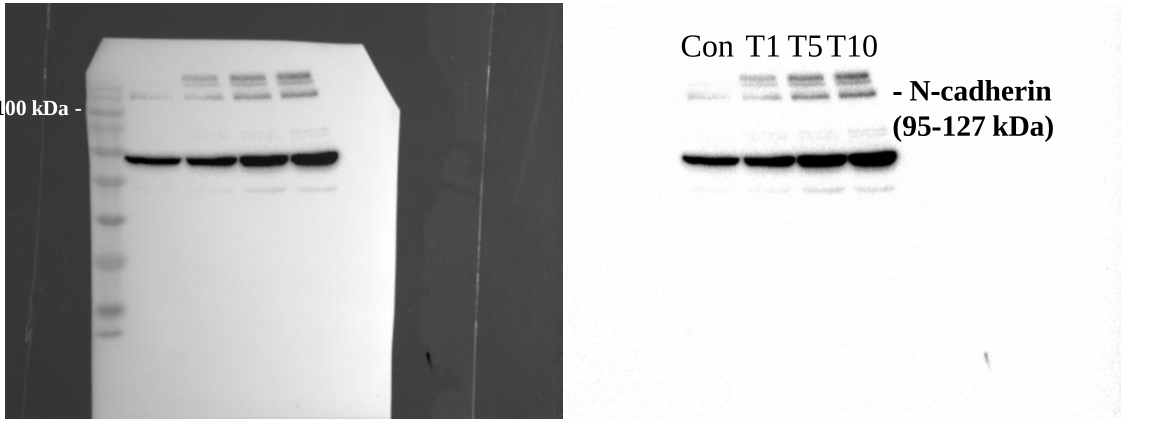

Figure 1E. A549_β-actin (host : Mouse)

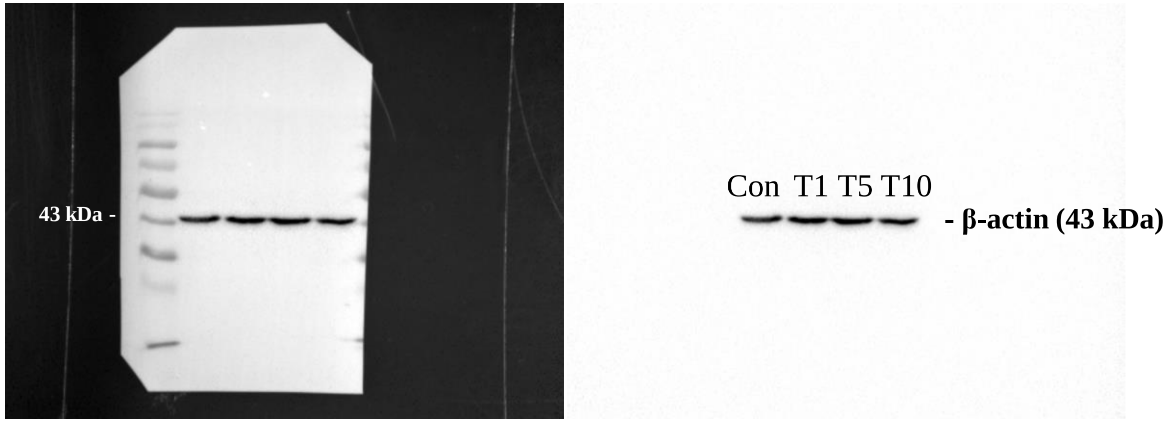

Figure 1E. BEAS-2B_β-actin (host : Mouse)

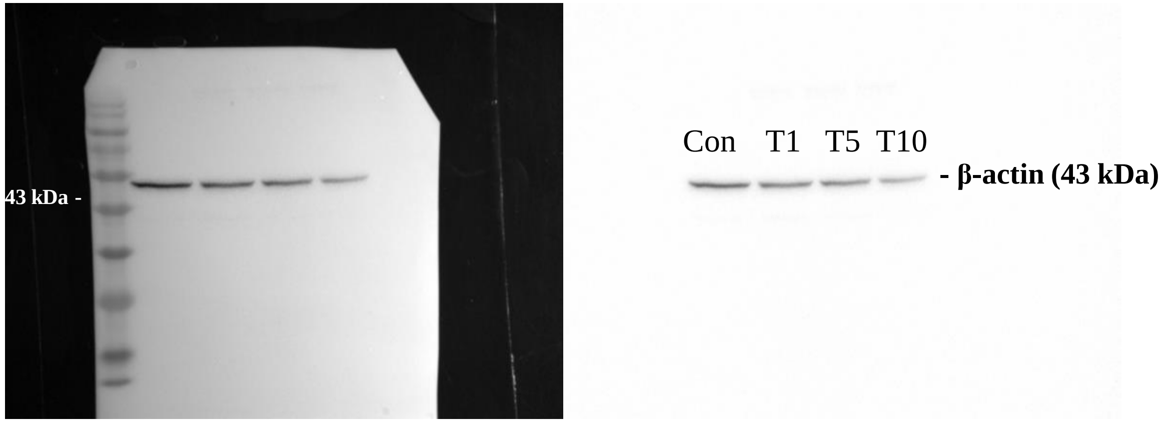

Figure 1. raw data

All of our protein quantifications were based on reference genes measured on the same blot.

Figure 1E. BEAS-2B_Fibronectin (host : Mouse)

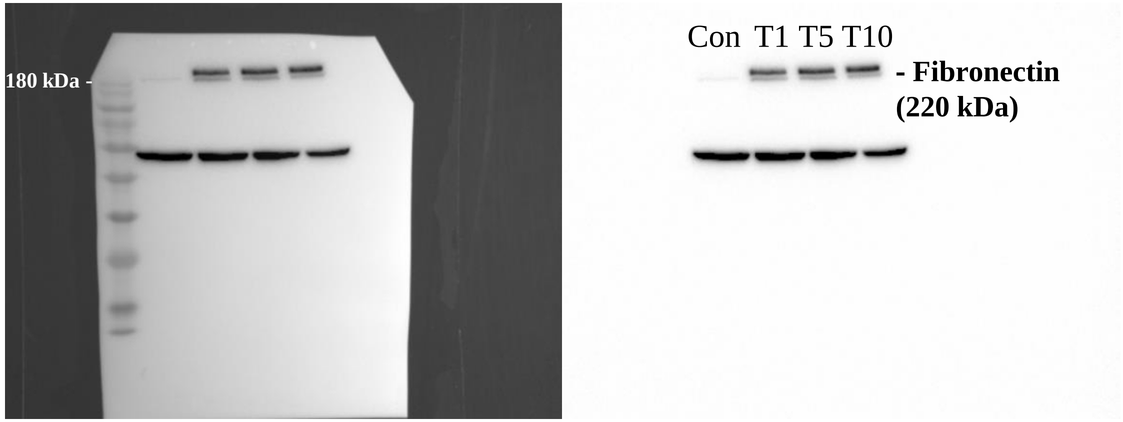

Figure 1E. BEAS-2B_Collagen1 (host : Rabbit)

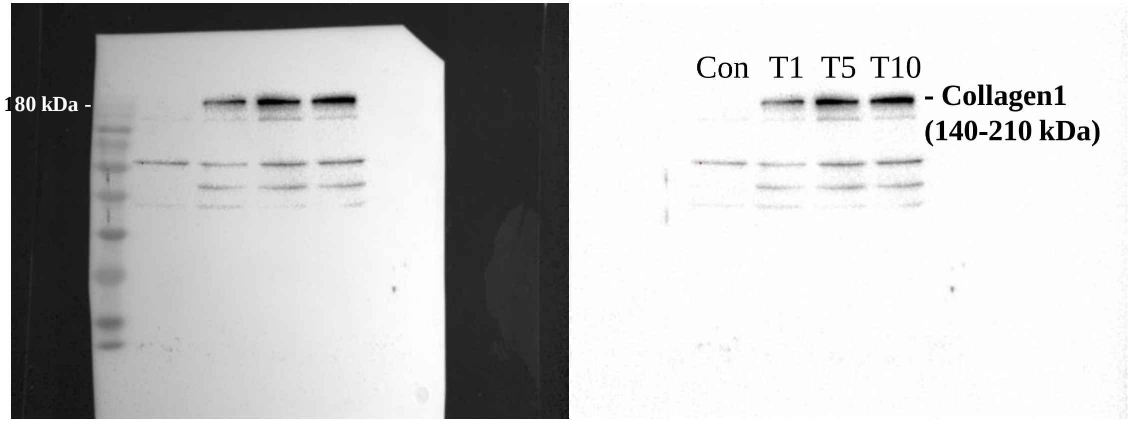

Figure 1E. BEAS-2B_β-actin (host : Mouse)

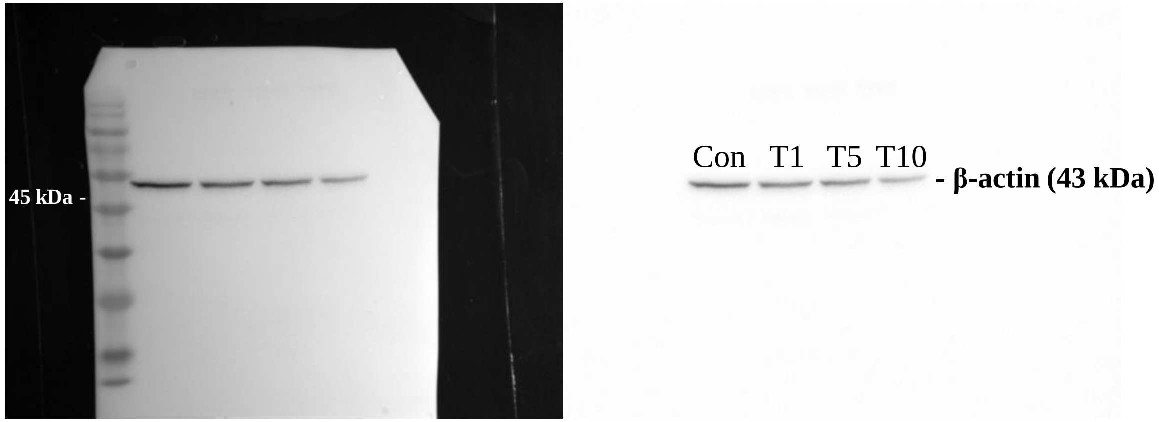

Figure 1E. BEAS-2B_β-actin (host : Mouse)

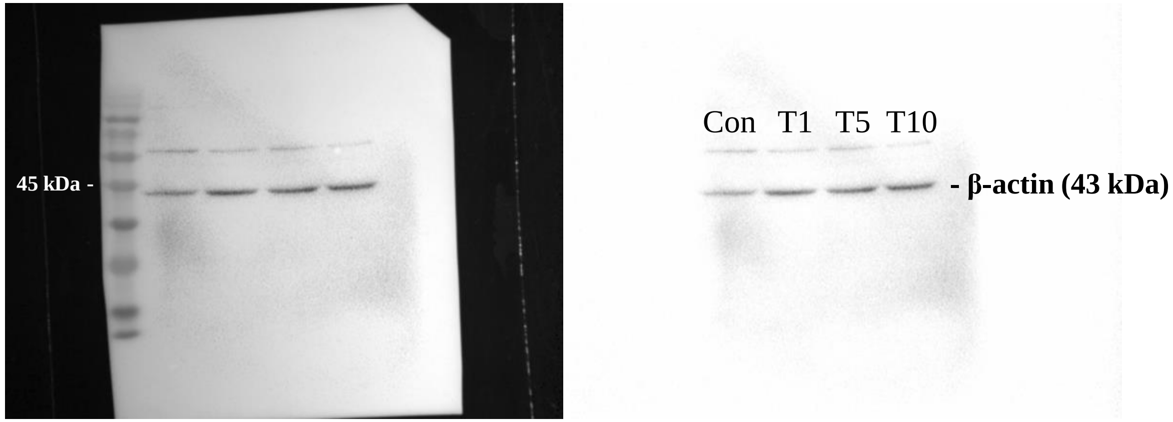

Figure 4. raw data

All of our protein quantifications were based on reference genes measured on the same blot.

Figure 4A. A549_p-SMAD3 (host : Mouse)

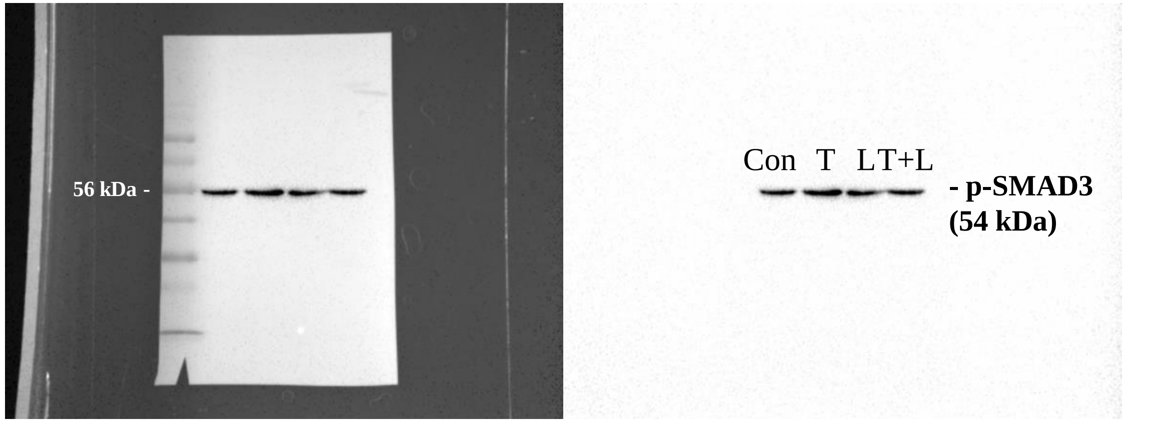

Figure 4A. A549_t-SMAD3 (host : Mouse)

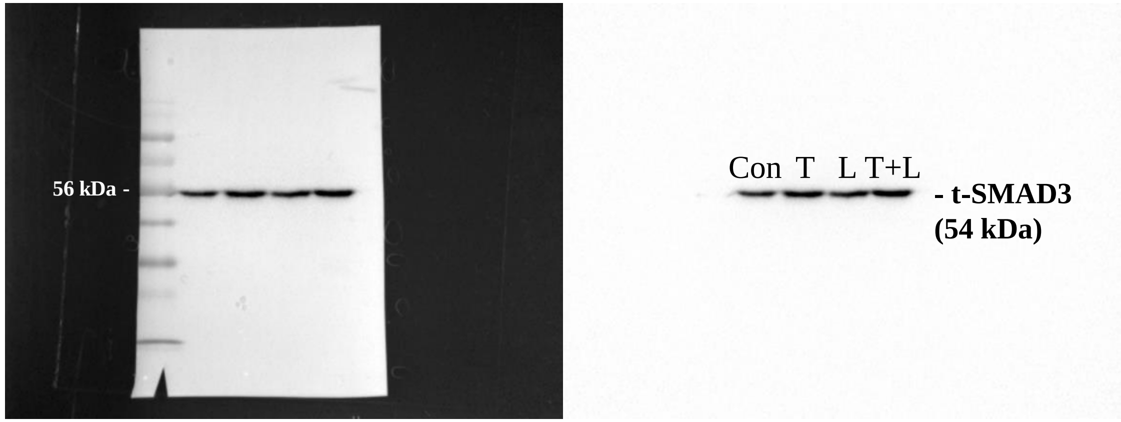

Figure 4A. A549_ β-actin (host : Mouse)

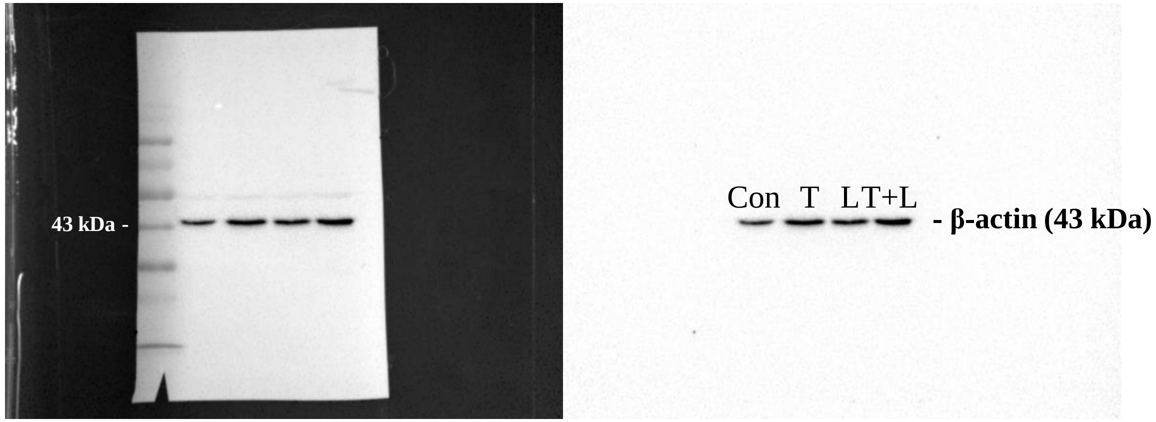

Figure 4. raw data

All of our protein quantifications were based on reference genes measured on the same blot.

Figure 4A. A549_Fibronectin (host : Mouse)

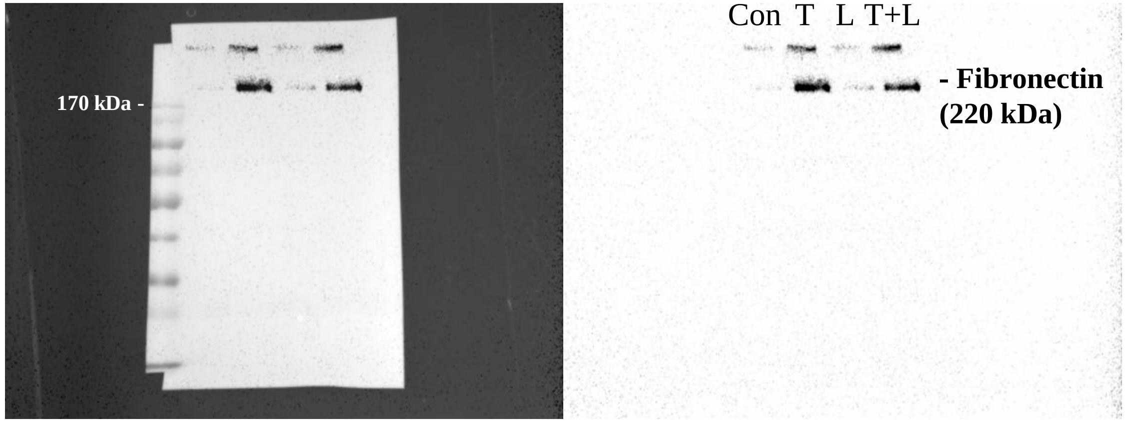

Figure 4A. A549_Collagen1 (host : Rabbit)

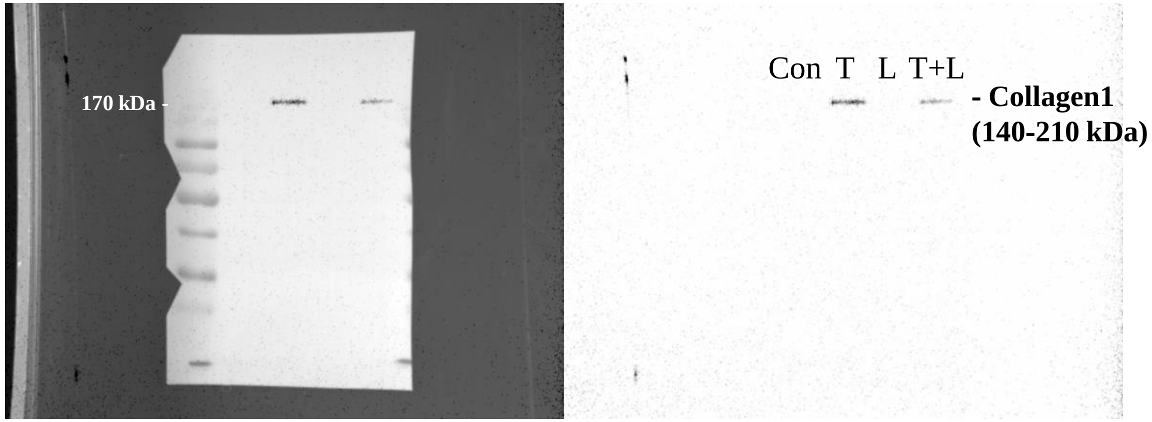

Figure 4A. A549_ β-actin (host : Mouse)

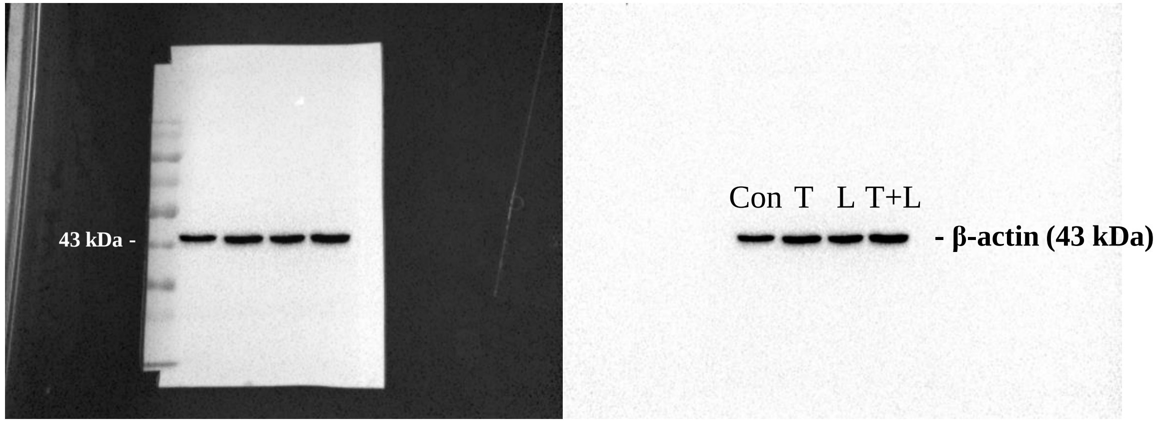

Figure 4A. A549_ β-actin (host : Mouse)

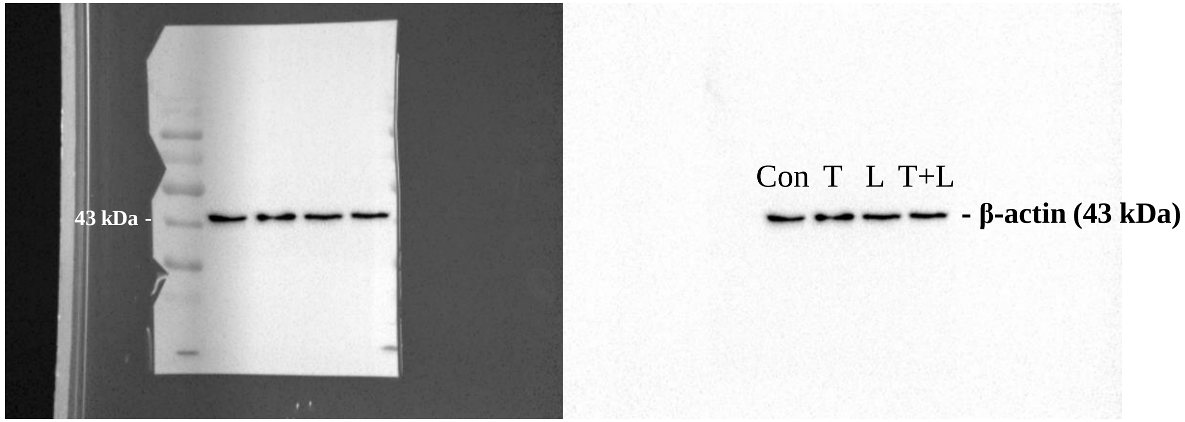

Figure 4. raw data

All of our protein quantifications were based on reference genes measured on the same blot.

Figure 4A. BEAS-2B _p-SMAD3 (host : Mouse)

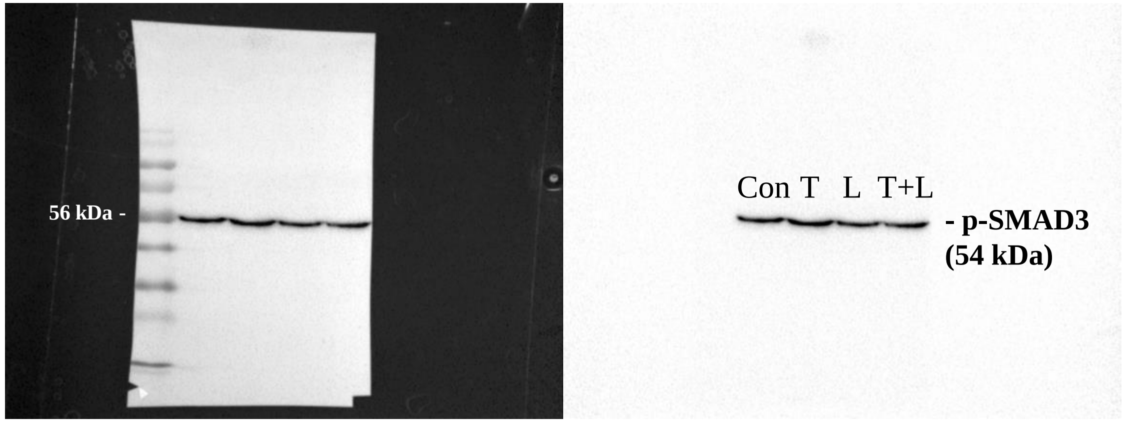

Figure 4A. BEAS-2B _t-SMAD3 (host : Mouse)

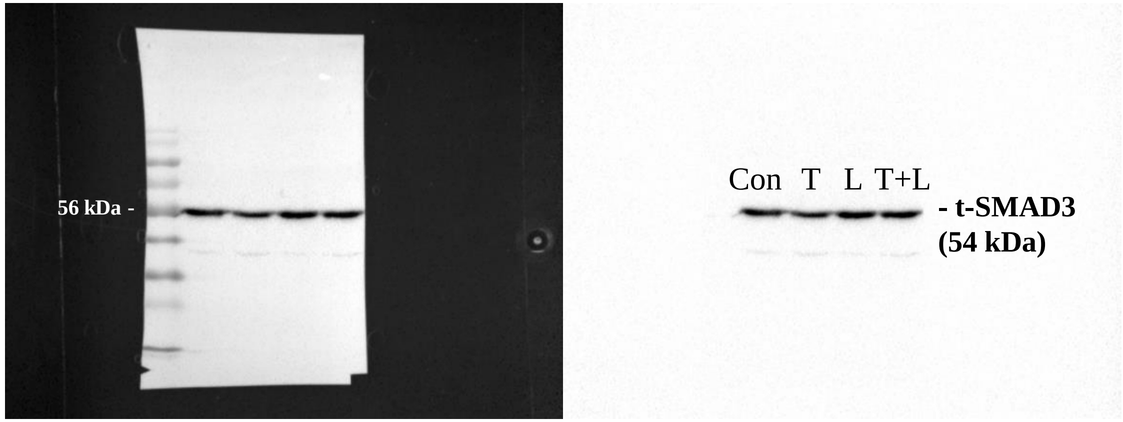

Figure 4A. BEAS-2B _ β-actin (host : Mouse)

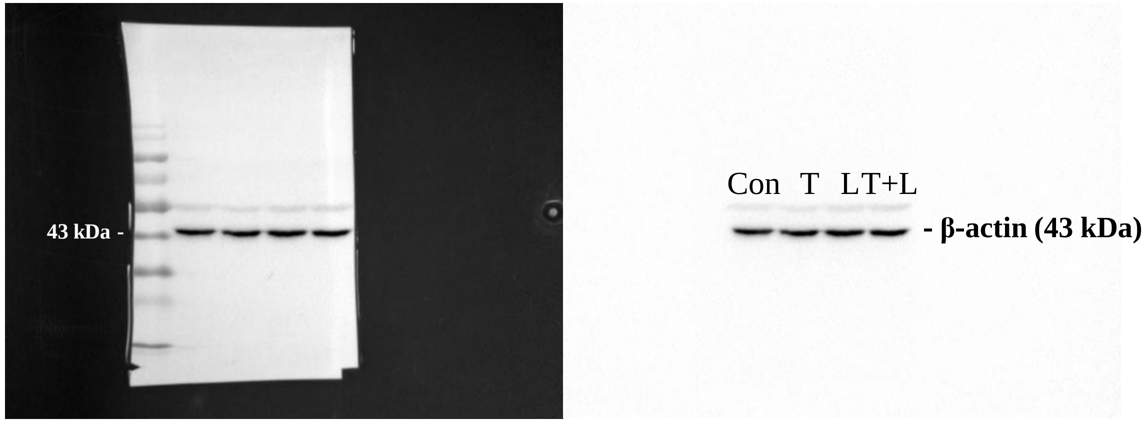

Figure 4. raw data

All of our protein quantifications were based on reference genes measured on the same blot.

Figure 4A. BEAS-2B_Fibronectin (host : Mouse)

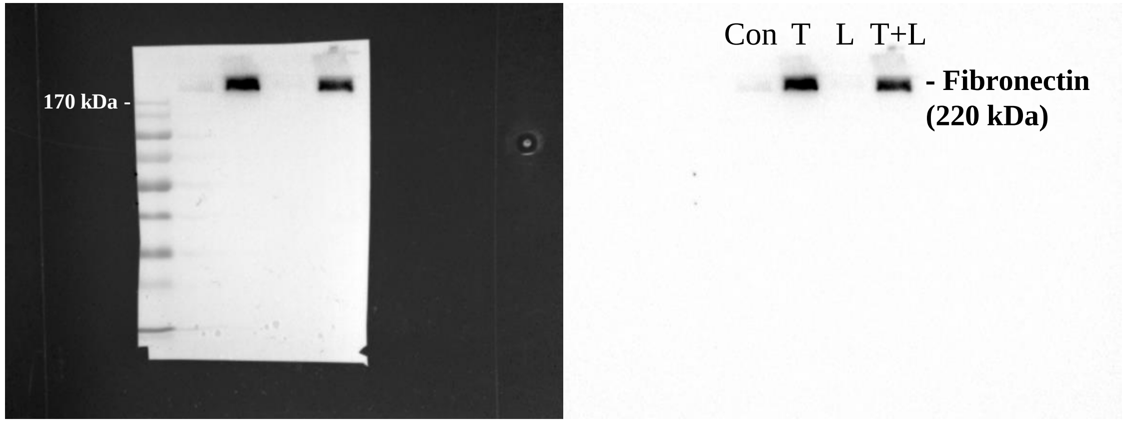

↑Same blot↓

Figure 4A. BEAS-2B _ β-actin (host : Mouse)

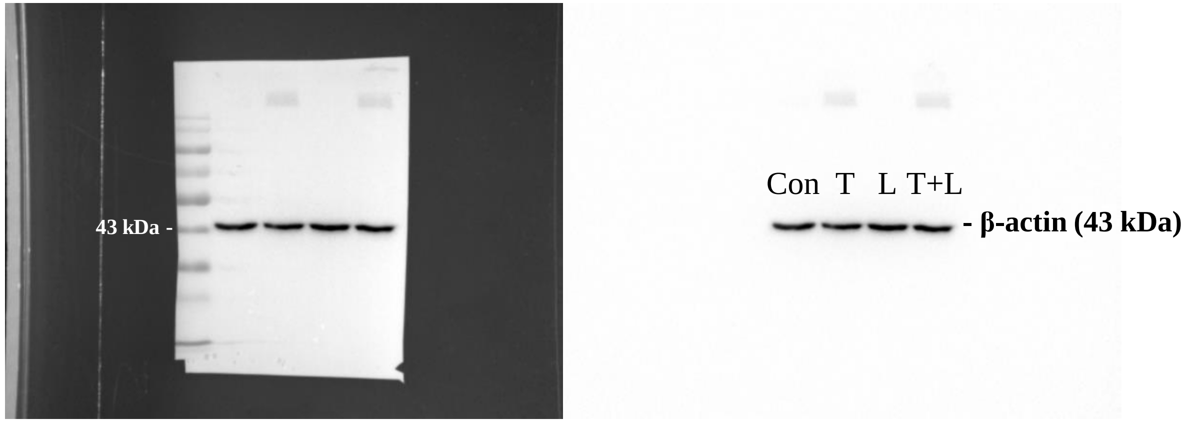

Figure 4A. BEAS-2B _Collagen1 (host : Rabbit)

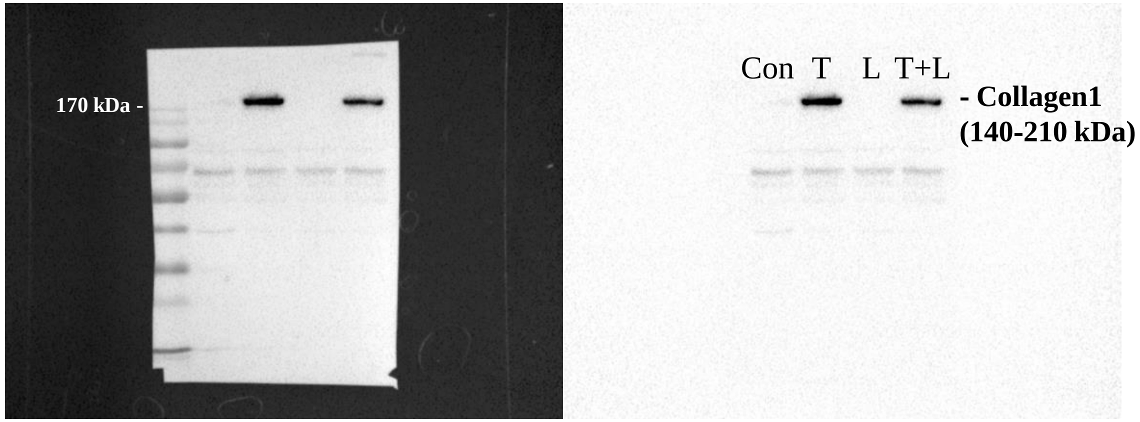

Figure 4A. BEAS-2B _ β-actin (host : Mouse)

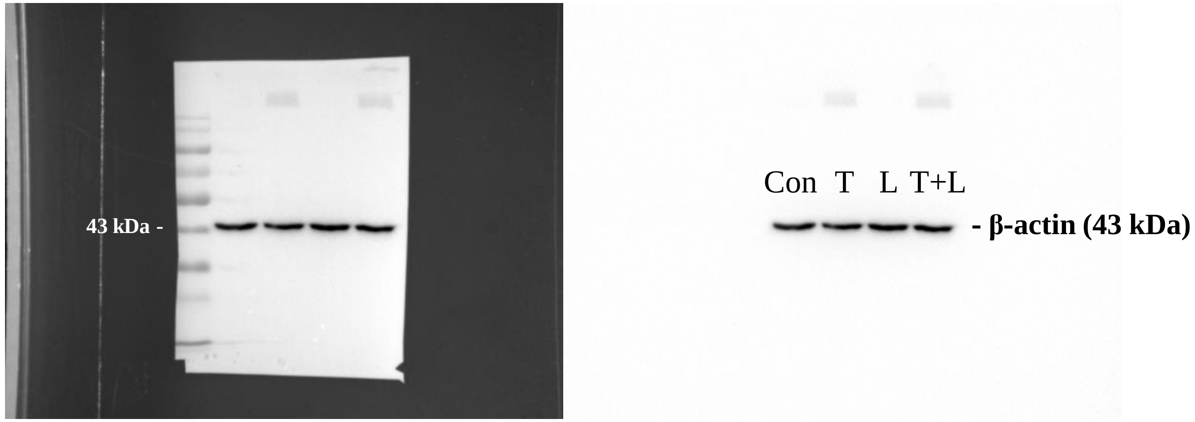

Figure 5. raw data

All of our protein quantifications were based on reference genes measured on the same blot.

Figure 5A. A549_THBS1 (host : Mouse)

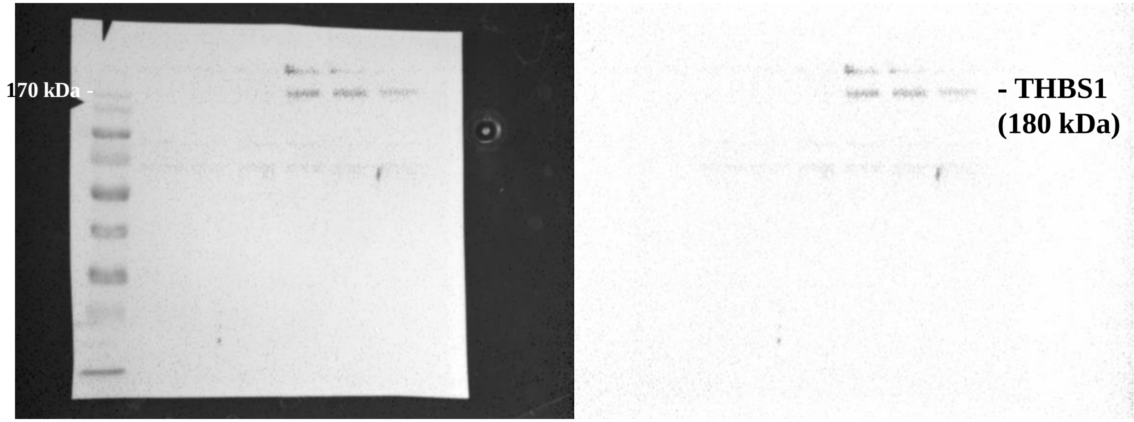

C / C+sc / C+mi / T / T+sc / T+mi

Figure 5A. A549_Collagen1 (host : Rabbit)

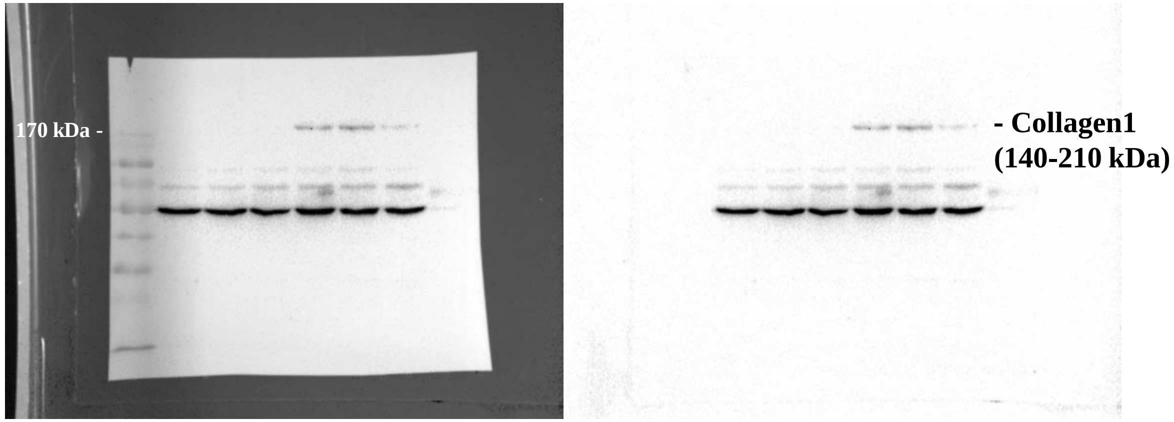

C / C+sc / C+mi / T / T+sc / T+mi

Figure 5A. A549_ β-actin (host : Mouse)

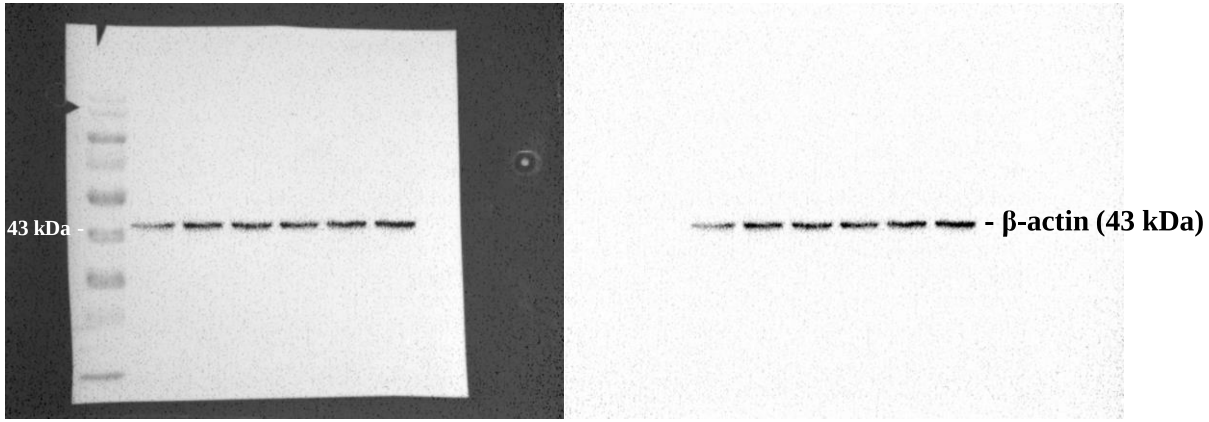

C / C+sc / C+mi / T / T+sc / T+mi

Figure 5A. A549_ β-actin (host : Mouse)

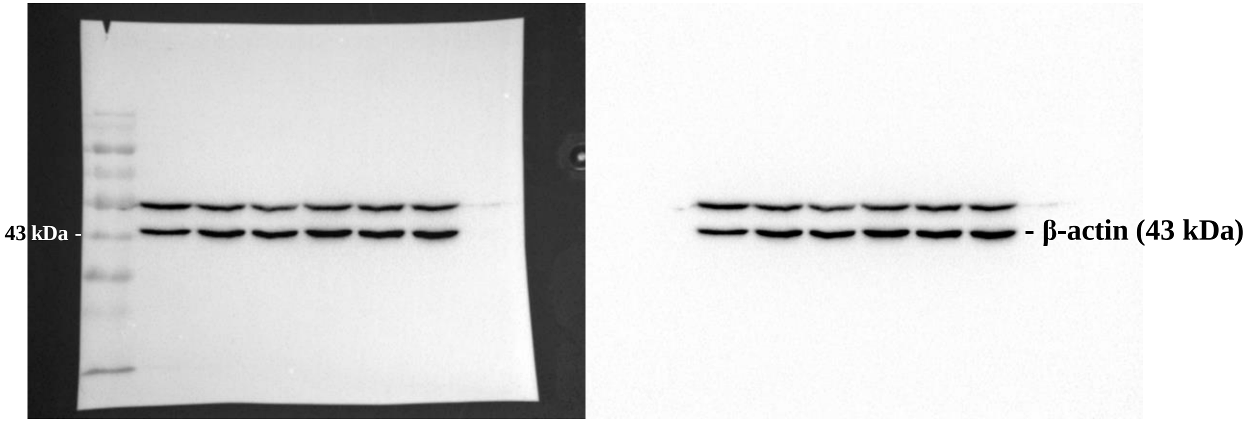

C / C+sc / C+mi / T / T+sc / T+mi

Figure 5. raw data

All of our protein quantifications were based on reference genes measured on the same blot.

Figure 5A. A549_Fibronectin (host : Mouse)

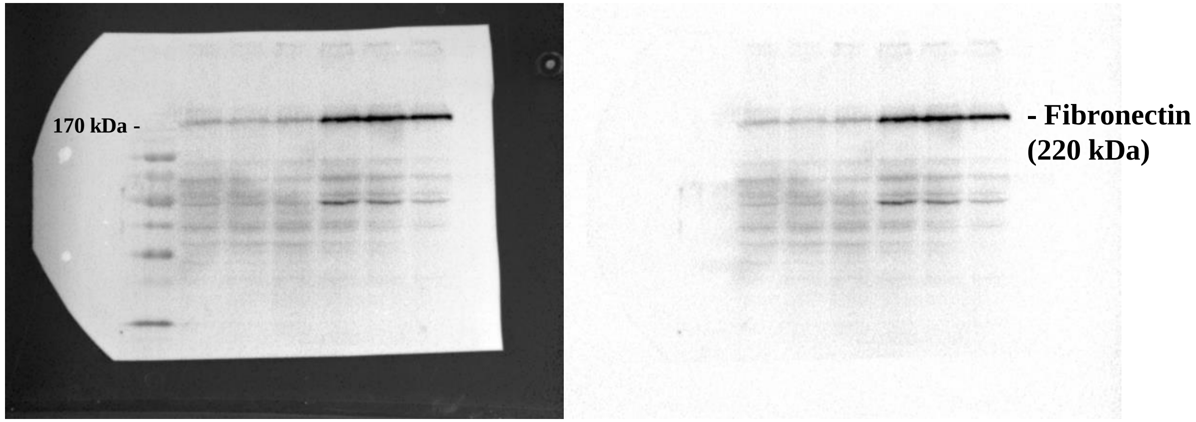

C / C+sc / C+mi / T / T+sc / T+mi

Figure 5A. BEAS-2B_THBS1 (host : Mouse)

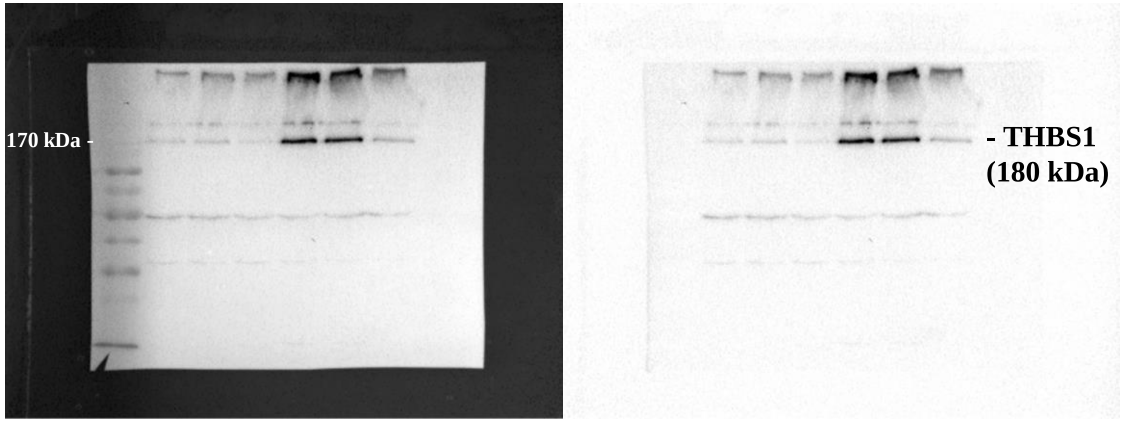

C / C+sc / C+mi / T / T+sc / T+mi

Figure 5A. A549_ β-actin (host : Mouse)

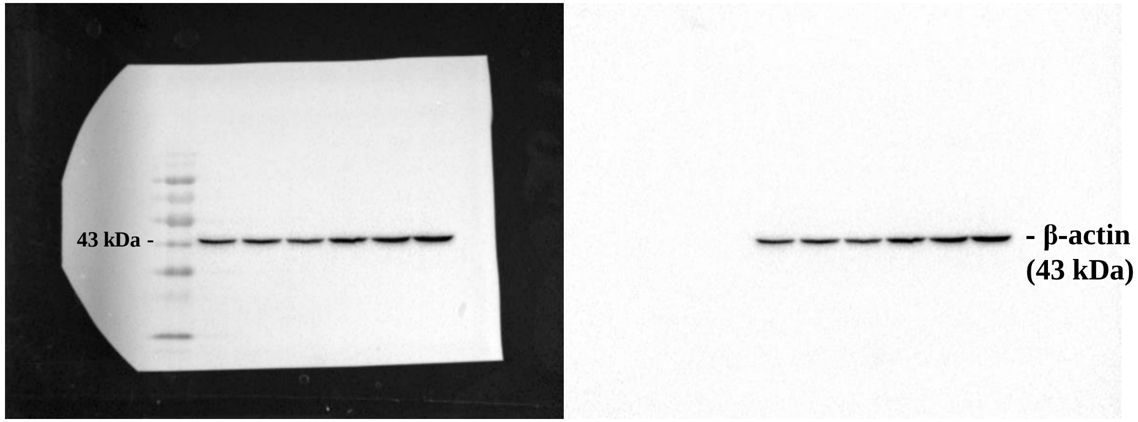

C / C+sc / C+mi / T / T+sc / T+mi

Figure 5A. BEAS-2B_ β-actin (host : Mouse)

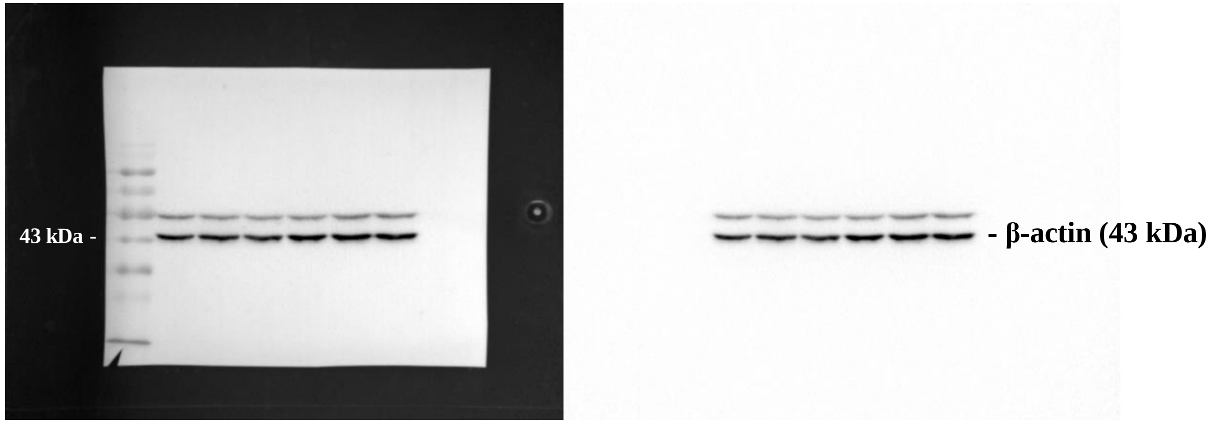

C / C+sc / C+mi / T / T+sc / T+mi

Figure 5. raw data

All of our protein quantifications were based on reference genes measured on the same blot.

Figure 5A. BEAS-2B_Collagen1 (host : Rabbit)

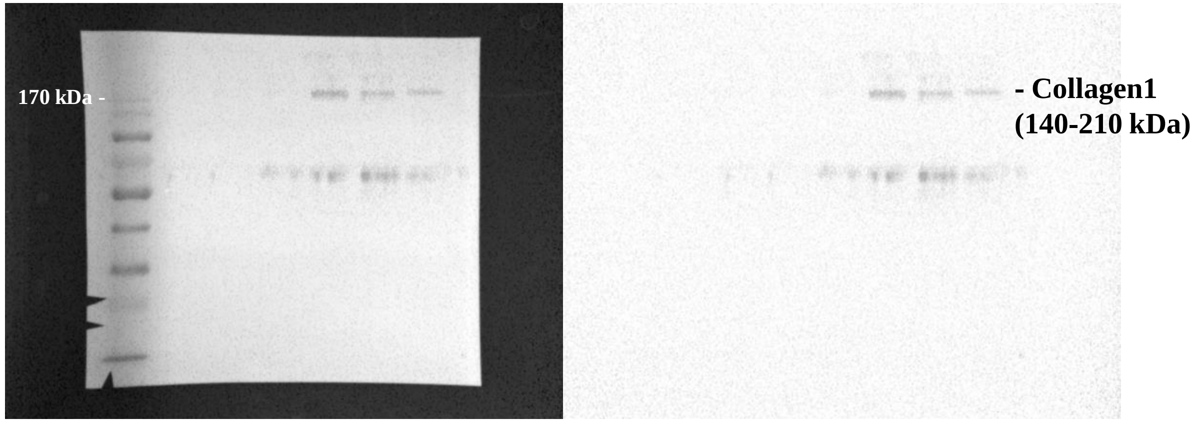

C / C+sc / C+mi / T / T+sc / T+mi

Figure 5A. BEAS-2B _Fibronectin (host : Mouse)

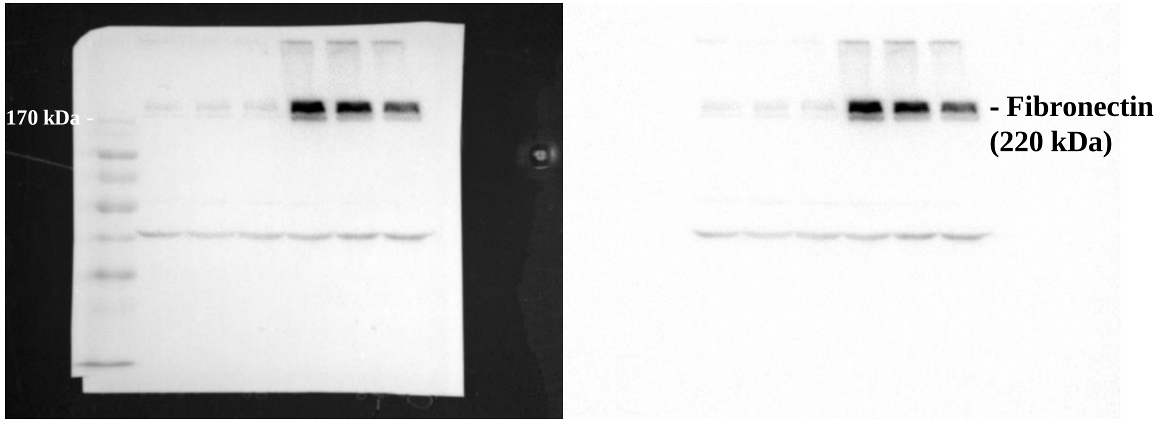

C / C+sc / C+mi / T / T+sc / T+mi

Figure 5A. BEAS-2B _ β-actin (host : Mouse)

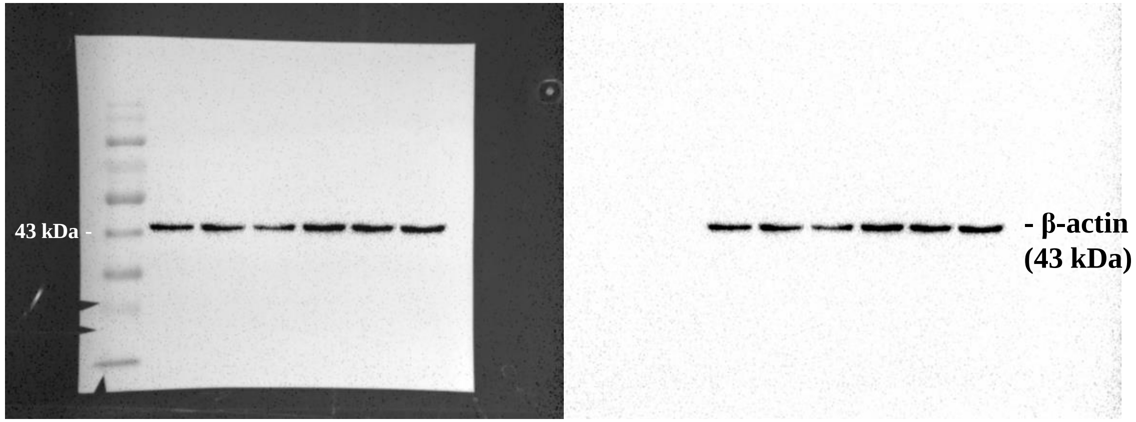

C / C+sc / C+mi / T / T+sc / T+mi

Figure 5A. BEAS-2B _ β-actin (host : Mouse)

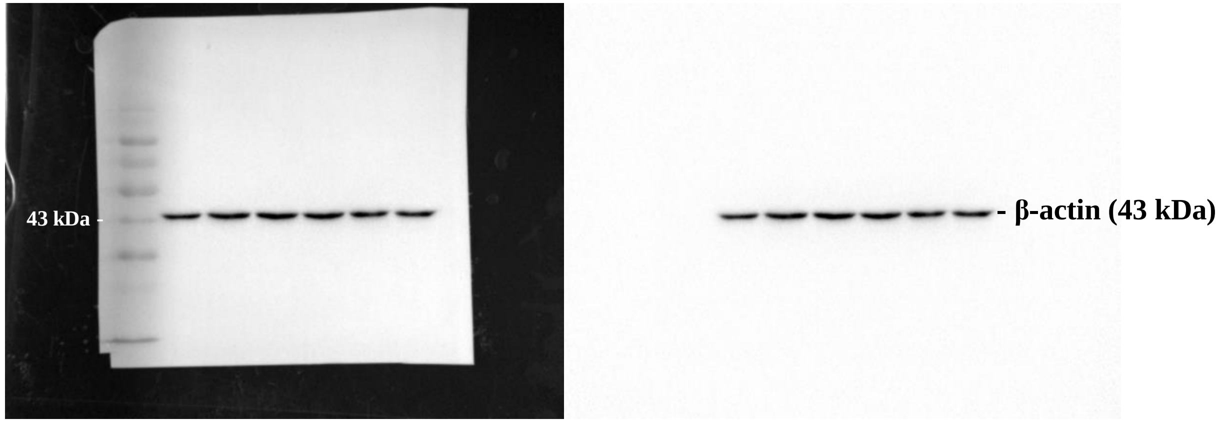

C / C+sc / C+mi / T / T+sc / T+mi

Figure 5. raw data

All of our protein quantifications were based on reference genes measured on the same blot.

Figure 5C. A549_THBS1 (host : Mouse)

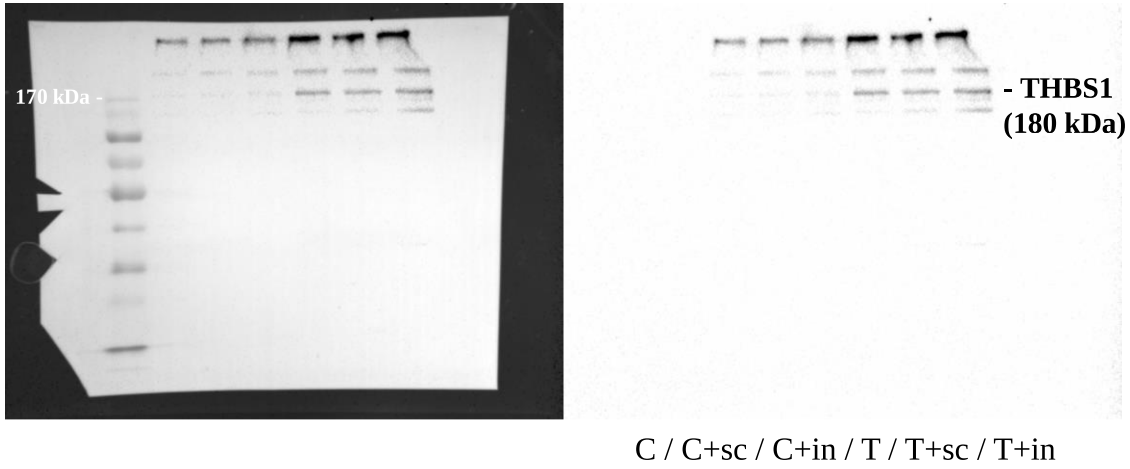

Figure 5C. A549_Vimentin (host : Mouse)

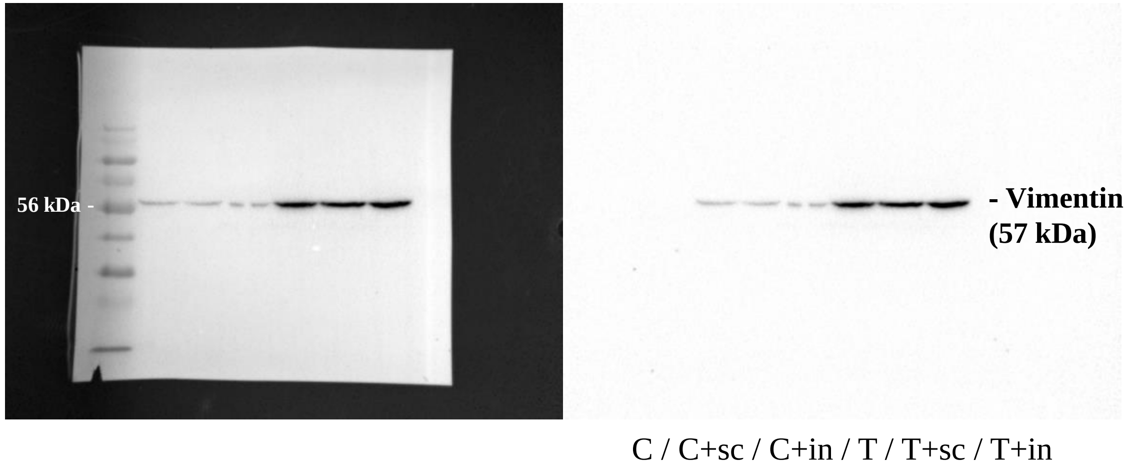

Figure 5C. A549_ β-actin (host : Mouse)

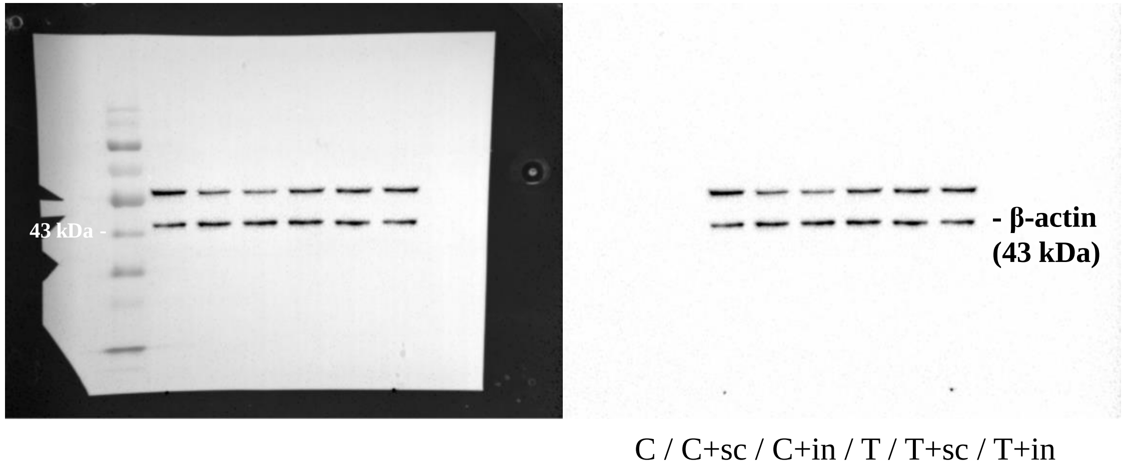

Figure 5C. A549_ β-actin (host : Mouse)

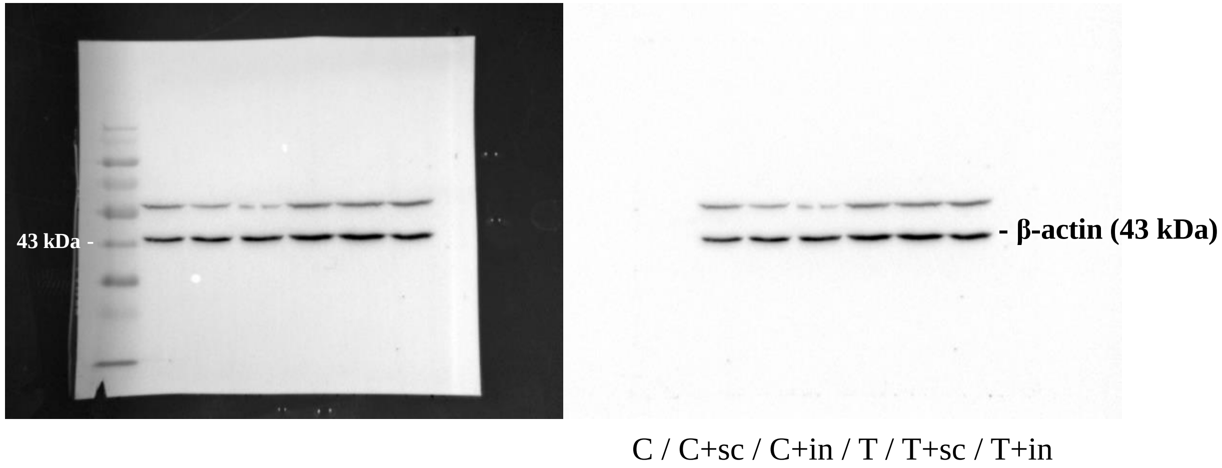

Figure 5. raw data

All of our protein quantifications were based on reference genes measured on the same blot.

Figure 5C. A549_Fibronectin (host : Mouse)

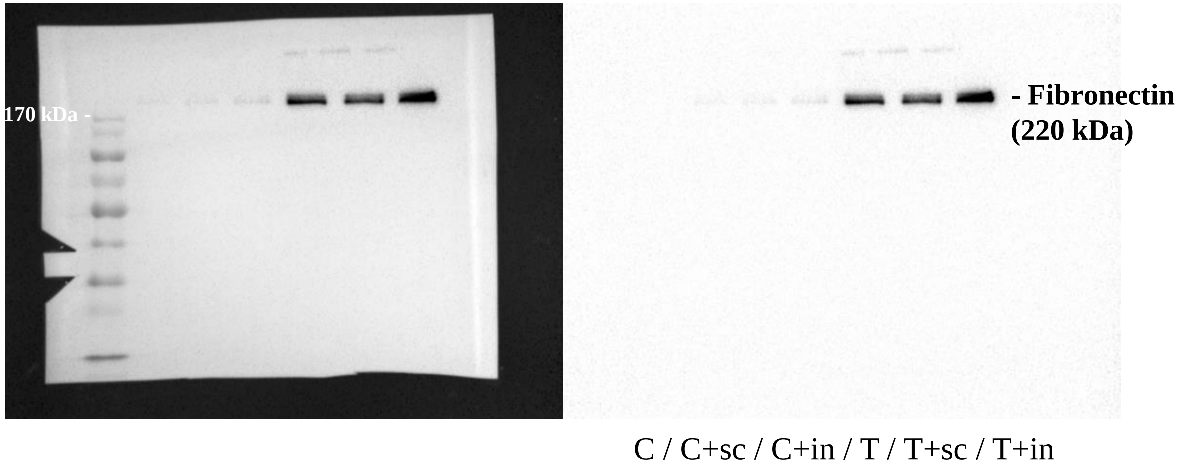

Figure 5C. BEAS-2B_THBS1 (host : Mouse)

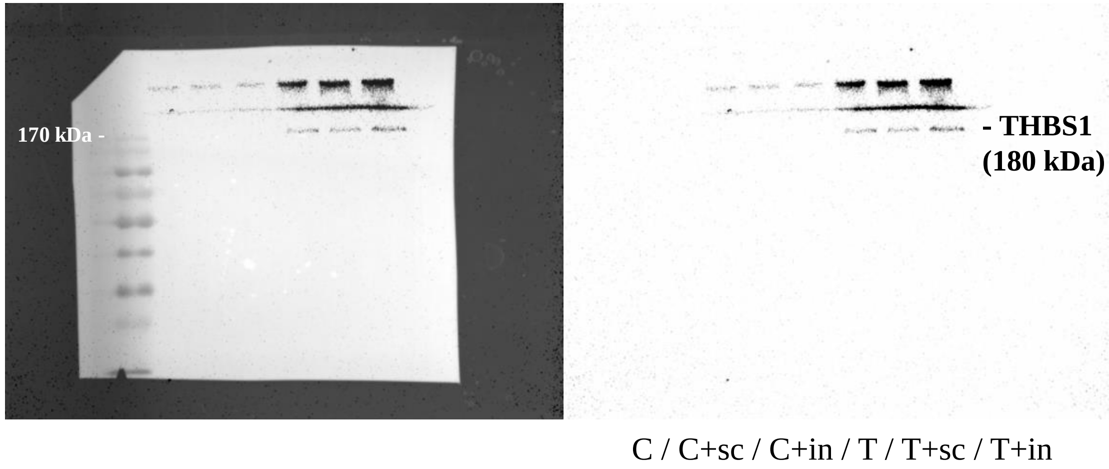

Figure 5C. A549_ β-actin (host : Mouse)

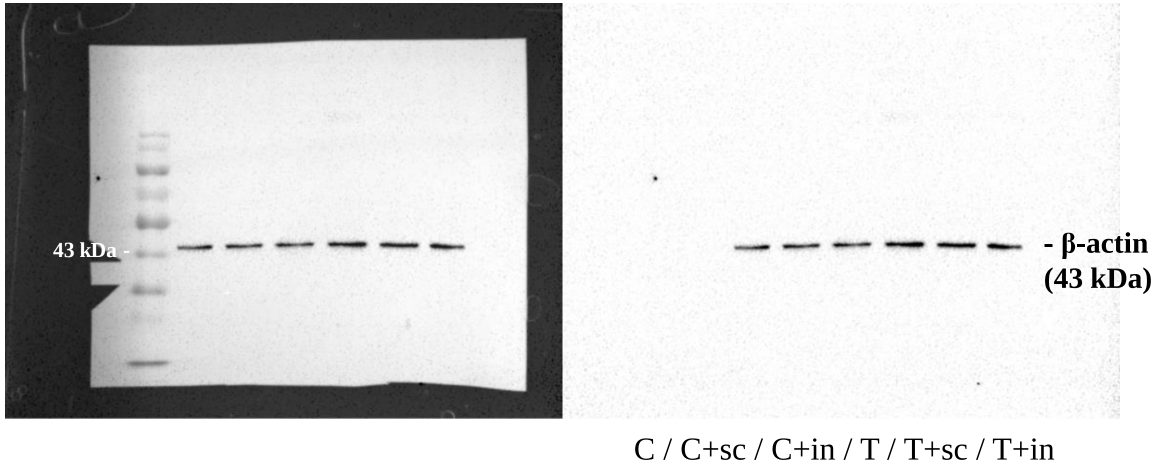

Figure 5C. BEAS-2B_ β-actin (host : Mouse)

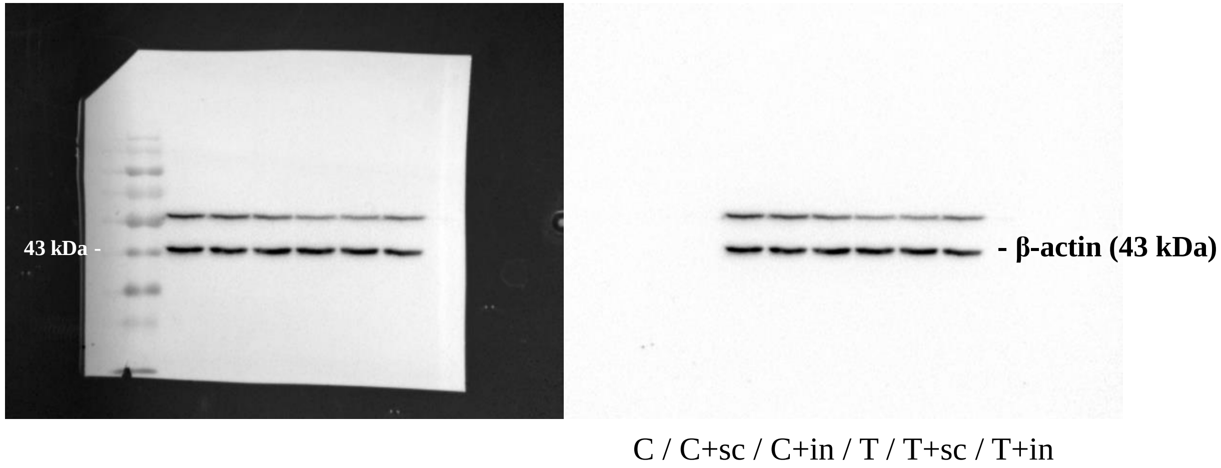

Figure 5. raw data

All of our protein quantifications were based on reference genes measured on the same blot.

Figure 5C. BEAS-2B_Vimentin (host : Mouse)

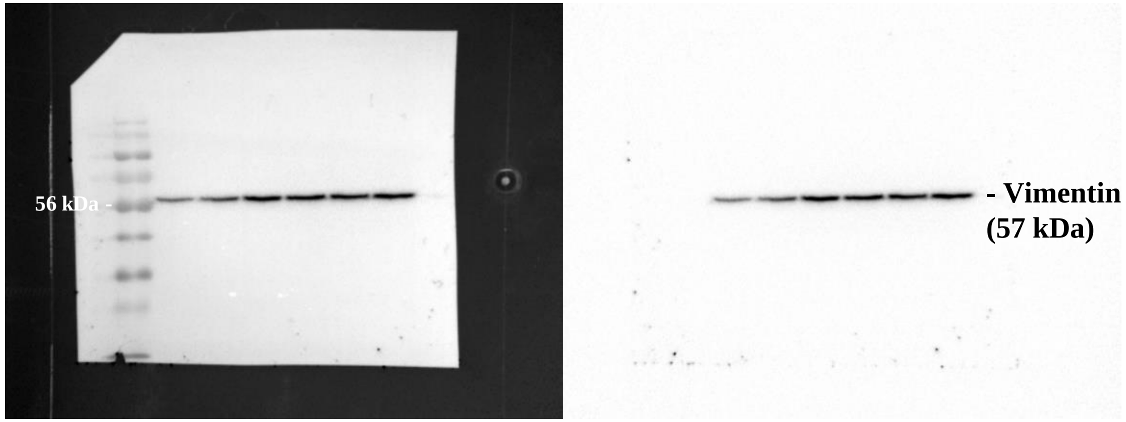

C / C+sc / C+in / T / T+sc / T+in

Figure 5C. BEAS-2B _Fibronectin (host : Mouse)

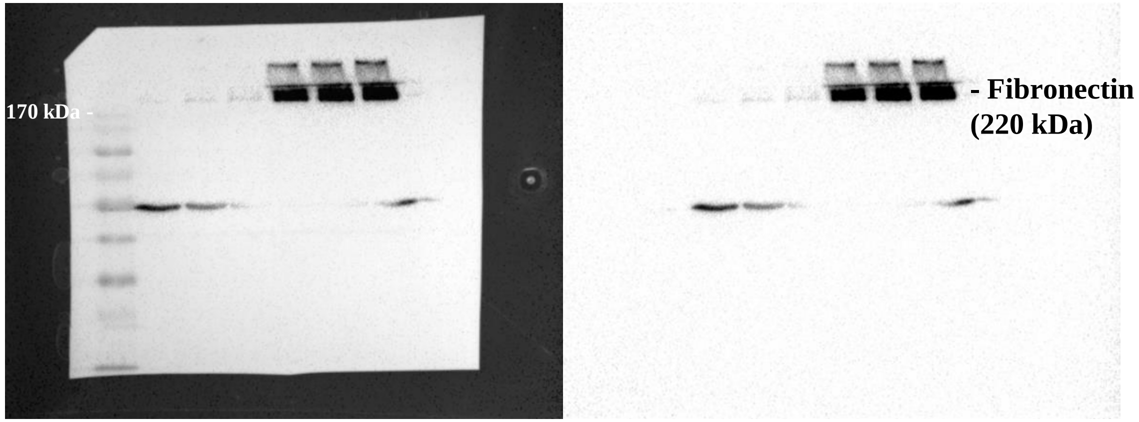

C / C+sc / C+in / T / T+sc / T+in

Figure 5C. BEAS-2B _ β-actin (host : Mouse)

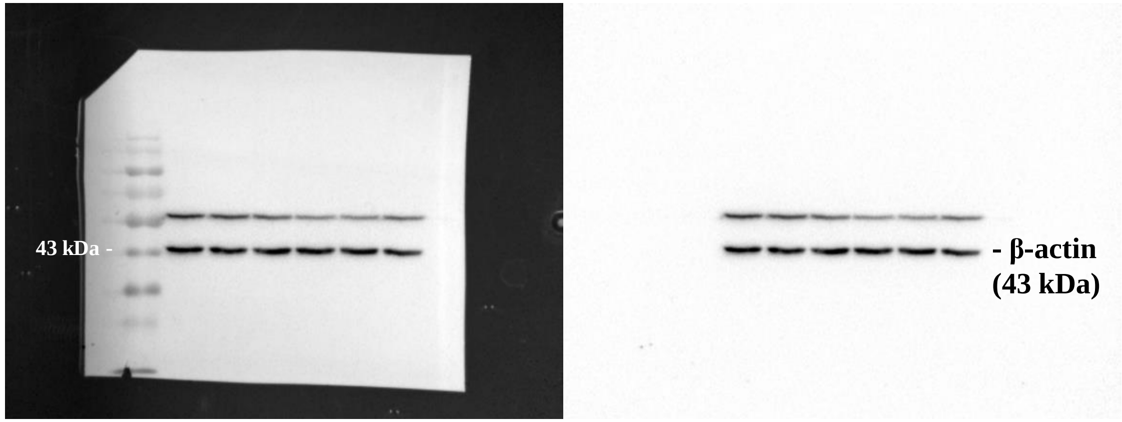

C / C+sc / C+in / T / T+sc / T+in

Figure 5C. BEAS-2B _ β-actin (host : Mouse)

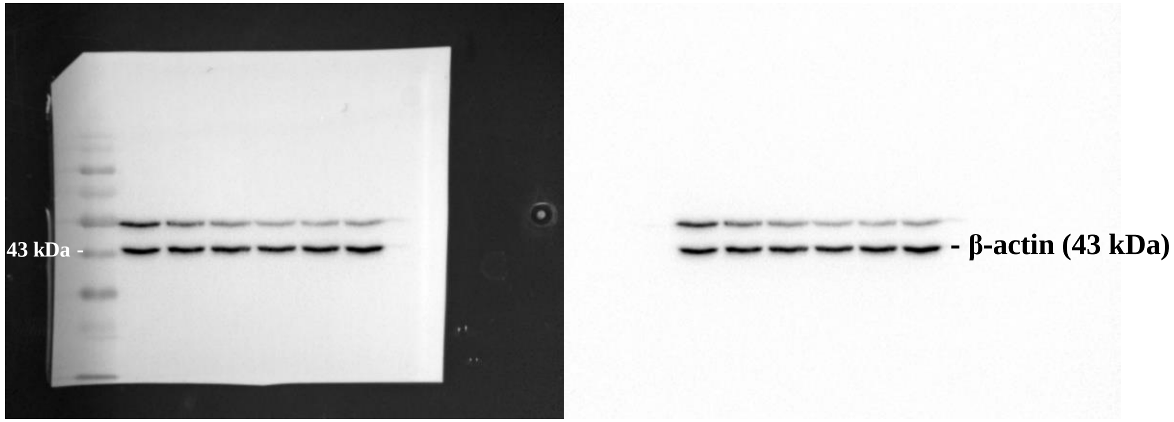

C / C+sc / C+in / T / T+sc / T+in
